# The Global Distribution and Drivers of Alien Bird Species Richness

**DOI:** 10.1371/journal.pbio.2000942

**Published:** 2017-01-12

**Authors:** Ellie E. Dyer, Phillip Cassey, David W. Redding, Ben Collen, Victoria Franks, Kevin J. Gaston, Kate E. Jones, Salit Kark, C. David L. Orme, Tim M. Blackburn

**Affiliations:** 1 Centre for Biodiversity and Environment Research, Department of Genetics, Evolution and Environment, University College London, London, United Kingdom; 2 Institute of Zoology, Zoological Society of London, Regent’s Park, London, United Kingdom; 3 Centre for Conservation Science and Technology, and School of Biological Sciences, University of Adelaide, Adelaide, South Australia, Australia; 4 Department of Zoology, University of Cambridge, Cambridge, United Kingdom; 5 Environment and Sustainability Institute, University of Exeter, Penryn, Cornwall, United Kingdom; 6 The Biodiversity Research Group, School of Biological Sciences, ARC Centre of Excellence for Environmental Decisions and NESP Threatened Species hub, The University of Queensland, Brisbane, Queensland, Australia; 7 Division of Biology, Imperial College London, Silwood Park, Ascot, Berkshire, United Kingdom; 8 Distinguished Scientist Fellowship Program, King Saud University, Riyadh, Saudi Arabia; 9 Centre for Invasion Biology, Department of Botany and Zoology, Stellenbosch University, Matieland, South Africa; University of Missouri at Saint Louis, United States of America

## Abstract

Alien species are a major component of human-induced environmental change. Variation in the numbers of alien species found in different areas is likely to depend on a combination of anthropogenic and environmental factors, with anthropogenic factors affecting the number of species introduced to new locations, and when, and environmental factors influencing how many species are able to persist there. However, global spatial and temporal variation in the drivers of alien introduction and species richness remain poorly understood. Here, we analyse an extensive new database of alien birds to explore what determines the global distribution of alien species richness for an entire taxonomic class. We demonstrate that the locations of origin and introduction of alien birds, and their identities, were initially driven largely by European (mainly British) colonialism. However, recent introductions are a wider phenomenon, involving more species and countries, and driven in part by increasing economic activity. We find that, globally, alien bird species richness is currently highest at midlatitudes and is strongly determined by anthropogenic effects, most notably the number of species introduced (i.e., “colonisation pressure”). Nevertheless, environmental drivers are also important, with native and alien species richness being strongly and consistently positively associated. Our results demonstrate that colonisation pressure is key to understanding alien species richness, show that areas of high native species richness are not resistant to colonisation by alien species at the global scale, and emphasise the likely ongoing threats to global environments from introductions of species.

## Author Summary

The introduction of alien species is one of the primary ways in which human actions are changing the environment. Alien species have been responsible for numerous global and local extinctions and are eroding the uniqueness of many natural environments. There is thus a basic need to understand which areas end up with more alien species. Here, we use a major new global database on the distribution of alien birds to show, first, how patterns in the number of species introduced to a location (colonisation pressure) have changed over time. We show that historical introductions were driven largely by European, and especially British, colonialism. However, the rate of bird introductions is increasing, with shifts in the locations of origin and introduction of species probably driven by the cage bird trade. We then combine information on where bird species have been introduced with a global map of alien bird species richness to identify the main drivers of richness. We show that colonisation pressure is the strongest predictor of alien bird species richness, but that there are other anthropogenic and environmental drivers. Most notably, once colonisation pressure has been accounted for, alien bird species richness is higher in areas where native bird species richness is higher.

## Introduction

The number of species naturally inhabiting a location (native species richness [NSR]) is ultimately driven by the combined processes of speciation, extinction, and immigration, and proximately by the suite of environmental, ecological, historical, and evolutionary factors that determine the interplay of these processes [1]. An important feature of the Anthropocene is the extent to which human activities have enhanced immigration [2], such that species are being intentionally or accidentally transported and introduced to areas well beyond the biogeographic barriers that normally prevent their spread, and at unprecedentedly high rates [3]. These species (here termed alien) may establish viable populations and subsequently spread in their new locations (a process termed invasion) [4], altering local and regional-scale species richness as well as patterns of species turnover across areas (i.e., alpha, gamma, and beta diversity, respectively) [5]. Alien species can adversely affect the native biota, driving populations and species to extinction [6], altering ecosystem function [7], and negatively impacting social and economic activities [8], providing significant impetus to understand drivers of the invasion process.

The starting point of the process of invasion by alien species is the translocation of individuals by human activities. However, the extent to which the resulting worldwide distribution of alien species richness (ASR) is entirely a consequence of human activities is unknown. ASR is the end point of a multistage process that involves the following: the transportation and introduction of species to areas where they do not naturally occur; the establishment of viable populations around the point of introduction; and the spread of established populations across the wider landscape [4]. Clearly, alien species richness is likely to be highly dependent in the first instance on the number of species relocated to a given area or location by human activities. This is known as colonisation pressure, which is calculated simply as the sum of the number of alien species introduced to a defined location (e.g., country, state, island, 1° grid cell) over some period of time (typically the full period over which introductions have occurred, although sometimes subsets of this period are specified), some subset of which will succeed in establishing an exotic population [9]. The ASR of a given region (or area) at a given point in time will therefore equal colonisation pressure minus the number of those introductions that fail to establish, but plus the number of alien species that have spread into the region or area having been introduced elsewhere. Alien species richness is therefore expected *a priori* to be a positive function of colonisation pressure [9]. It is also likely to depend on other anthropogenic factors such as the proximity of an area to locations of introduction and the amount of time that introduced species have had to establish and spread (S1 Table).

Alien species richness may also be influenced to some degree by the same natural processes that determine native species richness [10]. Natural processes are expected to influence which introduced populations succeed (or fail) in establishing, and which species spread into novel locations from other introduction sites. These components of alien species richness are likely to be functions of the abiotic environment, biotic interactions (primarily with native species because typically ASR < NSR; S1 Table), and stochasticity [11,12]. As a result, they will demonstrate structured spatial variation. However, the relative interplay of these different natural processes on alien species richness remains poorly known [3]. The primary reason is that for most alien taxa, we have very limited information on colonisation pressure, meaning that it is impossible to determine accurately the form of its expected effect on alien species richness. This omission is a fundamental barrier to a mechanistic understanding of other drivers of alien species richness. For example, an observed negative association between native and alien species richness could be evidence of biotic resistance to the spread of alien species (S1 Table), but equally could simply reflect higher colonisation pressure in areas of lower native species richness. Thus, determinants of variation in alien species richness cannot meaningfully be analysed without knowledge of variation in colonisation pressure.

Here, we present what is, to our knowledge, the first global analysis of the determinants of alien species richness that takes into account colonisation pressure. Our study uses birds (class Aves) as a model system and is underpinned by a database comprising 27,723 distribution records for 971 alien bird species worldwide (see Methods). Uniquely, for this species group, the high quality of our collated data means that our database includes relatively detailed information on where species have been introduced (both successes and failures). This data quality allowed us to quantify spatial and temporal variation in the key variable of colonisation pressure at a range of spatial and temporal scales and to test factors that underpin this variation. We show that the locations of origin and introduction, and the identities, of bird species introduced in the historical period (1500–1903 AD) were largely determined by planned liberations associated with European (mainly British) colonial expansion, whereas recent introductions (1983–2000 AD) were largely unplanned and associated with increased levels of economic activity. We then combine information on colonisation pressure with abiotic, biotic, and anthropogenic variables to identify which of the hypothesised determinants influence worldwide variation in alien species richness (S1 and S2 Tables). We show that global patterns of alien species richness in birds are determined by both human and biotic factors. Our results reveal that attempts to model alien species richness at regional scales without data on colonisation pressure have problems inferring causality due to uncertainty over whether drivers are acting on colonisation pressure or alien species richness.

## Results

### Spatial and Temporal Variation in Alien Bird Introductions

**Fig 1:**
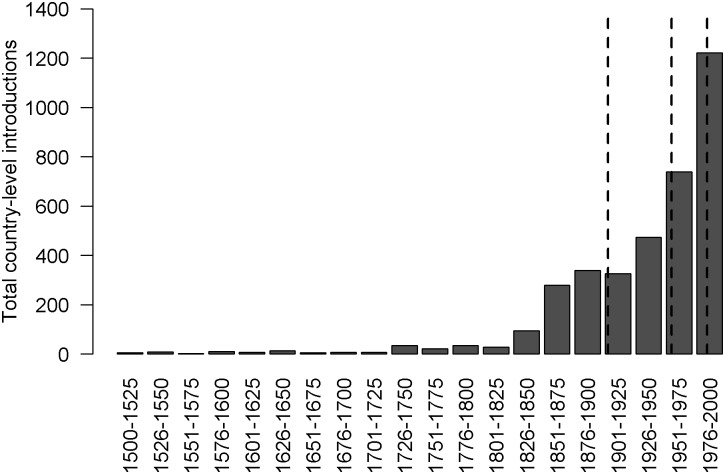
The total number of species introduced at a country level (i.e., species x country introductions) during the period 1500–2000 AD. The vertical dotted lines show the approximate locations of the divisions between the four quartiles of introduction; one quarter of all dated introductions in the period 1500–2000 lie to the left of the left-most dotted line, and one quarter lie to the right of the right-most dotted line.

We censored introduction data to the period 1500–2000 AD, because 1500 is a standard cutoff point for studying biological invasions [13,14], and introductions occurring after the year 2000 may not yet have entered the literature. Our analysis of introduction drivers covers a total of 3,661 alien bird introduction records (first known occurrence of a given species in a given country), which were distributed over time as shown in Fig 1. These 3,661 records were split into four quartiles, on the basis of introduction date, for analysis.

**Fig 2:**
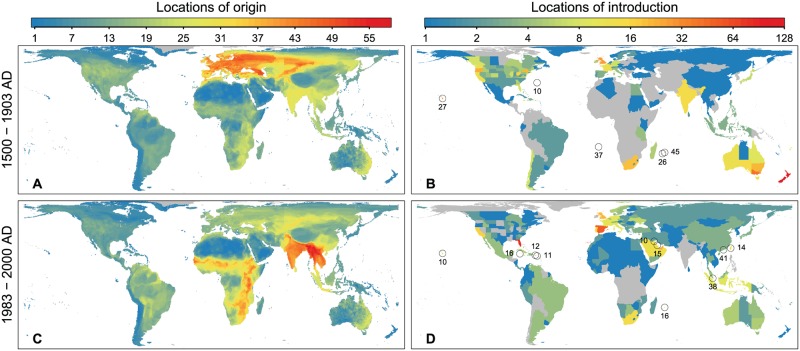
Locations of origin and introduction for bird species with introduced populations. **(A)** The overlap in distribution of native ranges (i.e., richness) of the alien bird species introduced in the first quartile of population introductions ordered by date (1500–1903 AD). **(B)** Locations of first introduction records for alien bird populations (i.e., state or country-level colonisation pressure) in the first quartile. **(C)** The overlap in distribution of native ranges of the alien bird species introduced in the fourth quartile of introductions ordered by date (1983–2000 AD). **(D)** Locations of first introduction records for alien bird populations (i.e., state or country-level colonisation pressure) in the fourth quartile. In **(A)** and **(C)**, cold colours represent lower native richness of bird species that were introduced in the period, warm colours represent higher richness, and grey areas are those not covered by the native ranges of any introduced species. In **(B)** and **(D)**, cold colours represent countries with low numbers of alien bird population introductions, warm colours represent countries with higher numbers, and grey countries are those without any record of alien bird populations having been introduced during this period. Numbers associated with circles record the number of alien bird species introductions to specific islands that would otherwise not be clearly visible on these maps.

**Fig 3:**
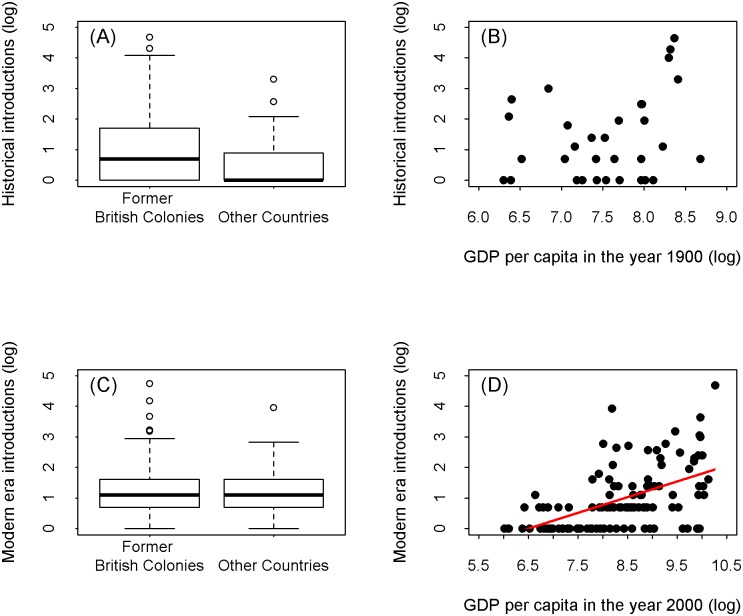
The relationship between the number of introduced bird species, colonial history, and GDP. The number of alien bird introductions in former British colonies and other countries for **(A)** the first quartile of dated bird introductions (1500–1903 AD) (Wilcoxon rank sum test: statistic = 5,557, *p* < 0.01) and **(C)** the fourth quartile of dated bird introductions (1983–2000 AD) (Wilcoxon rank sum test: statistic = 4,424, *p* = 0.99), ranked by date. The relationship between log number of species introduced to a country and its log GDP per capita for **(B)** the first quartile of dated bird introductions (1500–1903 AD; GDP data from 1900 AD) (n = 32; F_1, 30_ = 2.72, adj R^2^ = 0.05, *p* = 0.1), **(D)** the fourth quartile of dated bird introductions (1983–2000 AD; GDP data from 2000 AD) (n = 118; F_1, 116_ = 50.87, adj R^2^ = 0.29, *p* < 0.001; regression line shown in red). A constant of 1 was added to each variable prior to log transformation.

The rate and drivers of alien bird introductions altered markedly over the period 1500–2000 AD. The first quartile of bird introductions ordered by date spans 403 y (1500–1903 AD; see Methods), during which a total of 245 species were introduced to 167 countries (922 introductions in total). In contrast, the fourth quartile covers just 17 y (1983–2000 AD), with a total of 324 species introduced to 235 countries (935 introductions in total). The rate increased sharply around the middle of the nineteenth century, such that 69.3% of first-quartile introductions were after 1850 (Fig 1). This increase coincides with the founding of the first Acclimatisation Societies [15,16], which largely drove introductions in this period. These organisations were specifically aimed at promoting introductions of beneficial species, such as game, and were especially prevalent in the then British territories [15,16]. Thus, families with the most introduced species are the main game-bird families—Anatidae (duck, geese, swans), Phasianidae (pheasants, grouse, partridge) and Columbidae (pigeons, doves) (S3 Table). Species in the first quartile of introductions were more likely to originate from Europe (Fig 2A, S4 Table). Early introductions were concentrated in fewer countries than expected (observed: 167; expected median [range]: 212 [189–231], *p* < 0.0002; see Methods), and these countries were more likely to be constituents of the then British Empire (Figs 2B and 3A).

The rate of bird introductions increased again from around the middle of the twentieth century, such that half of all known bird introductions occurred after 1956 (Fig 1). Recent introductions (1983–2000 AD) can be better explained by globalisation and economic growth [17]; the number of introductions to a country in the fourth quartile is positively correlated with its per capita GDP (Fig 3D), but not whether or not the introduction was to a former British colony (Fig 3C). The sources of introduced species have shifted significantly over the twentieth century, away from Europe and to the Indian subcontinent, Indochina, and sub-Saharan Africa (Fig 2C, S1 Fig, S4 Table). Three of the four bird families with the most introduced species in this period include popular cage birds—Psittacidae (parrots), Estrildidae (soft-bill finches), Sturnidae, (mynas, starlings) (S3 Table)—reflecting a shift towards unplanned introductions (or releases) of species in the cage and pet trade [18]. Introductions are now spread across more countries than in the first quartile, and more countries than expected by chance (observed: 235; expected median [range]: 214 [194–235], *p* = 0.0002). The locations of introduction of alien birds in the most recent quartile reflect the geographic focus of the bird trade [18], with notable hotspots in the Far East (e.g., Singapore, Hong Kong, Taiwan), the Near East, Spain, and Florida (Fig 2C and 2D).

### Determinants of Alien Bird Species Richness

**Fig 4:**
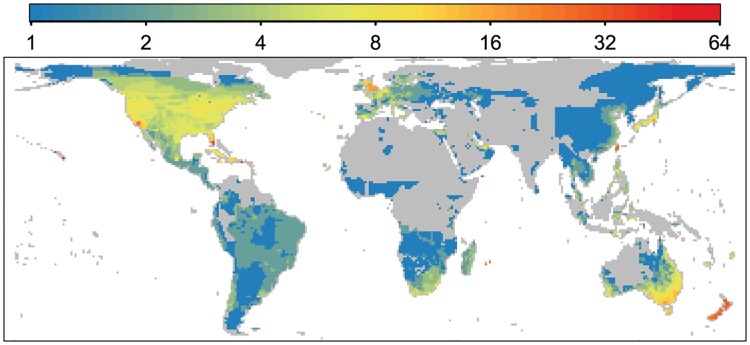
Global map of alien bird species richness. The figure is based on the 362 bird species with established alien distributions recorded with sufficient detail to map (see Methods). Colder colours indicate lower bird species richness, while warmer colours represent higher richness. Grey areas are those where there are no established bird populations.

The establishment (or failure) and subsequent spatial spread of populations of introduced bird species modifies the global patterns of introductions (Fig 2B and 2D, S1 Fig) to give rise to the current global distribution of alien bird species richness (Fig 4). As expected, colonisation pressure is a strong positive determinant of bird alien species richness and is, indeed, the variable most closely associated with alien species richness in our models (Table 1, S5 and S6 Tables, S2 Fig). As a result, the global map of alien bird species distributions shows that regions with high alien species richness tend to be located in temperate regions at midlatitudes (Fig 4); these are regions where former British colonies, rapidly developing countries, and countries with high per capita GDP are located, and where colonisation pressure has been concomitantly high (Fig 2B and 2D). Notable hotspots of alien bird species richness include areas in the United States (including the Hawaiian Islands), Caribbean, United Kingdom, Japan, Taiwan, Hong Kong, New Zealand, Australia, Persian Gulf States, and the Mascarene Islands. Alien species richness is also higher in areas with a longer history of introductions, with a significant positive effect of the number of years since the first bird introduction to a region, and shows a negative relationship with distance to a historic port (Table 1, S5 and S6 Tables, S2 Fig). These effects are independent of a measure of general human activity, the human footprint index (Table 1, S5 and S6 Tables, S2 Fig).

**Table 1:** Spatial correlates of alien bird richness.

Parameter	Estimate	S.E.	ΔwAIC
**Intercept**			
	-0.58	0.14	
**Anthropogenic**			
Colonisation pressure	0.050	0.0012	1206.8
(Colonisation pressure)^2^	-0.0003	1E–5	381.0
Time since introduction	0.0026	0.0002	153.4
(Time since introduction)^2^	-9.6E–7	6E–8	14.4
Distance to historic port	-9E–8	4E–8	12.2
**Environmental**			
Native Species Richness	0.0021	0.0001	320.2
(Native Species Richness)^2^	-1.9E–6	2E–7	123.0

The table presents the results of the minimum adequate model (MAM) for alien species richness in terms of the variables in S5 Table as described in the Methods (section xiv), and so includes only variables that improved model fit relative to a model excluding that variable (i.e., that reduced wAIC of the model by >4). Parameter estimates derive from fitting a Gaussian random field to the data to approximate the patterns of spatial autocorrelation in a Bayesian additive regression model inferred using INLA. Measures of goodness of fit for this model: wAIC = –13,839, conditional predictive ordinate (CPO) = 5,665.1, pseudo R^2^ = 98.6%. For comparison, fitting an intercept-only model gives wAIC = –11,610.6 and CPO = 4,457.5. S.E. = standard error. n = 10,258 grid cells. ΔwAIC = the change in wAIC relative to the MAM as a result of removing the variable, with larger positive changes for variables that more strongly influence the model.

The alien species richness of birds is not only a function of human historical factors but also shows significant imprints of the natural environment. Notably, there is a strong positive relationship between alien species richness and native species richness at the global scale (Table 1, S5 and S6 Tables, S2 Fig). Within the global regions to which alien bird species have been introduced or spread, areas with fewer native bird species tend also to house fewer aliens, with alien species richness peaking at mid to high levels of native species richness (S2 Fig). This may indicate that whatever drives native species richness may also drive alien species richness. Native species richness for a wide range of terrestrial taxa, including birds, tends to be higher in warmer, wetter regions [19], perhaps because these areas have higher levels of energy availability [20,21] or less physiologically stressful environments [22]. Native bird richness also tends to be higher in regions with greater elevational ranges [19]. Univariate analysis suggests that alien species richness also follows these trends, as regions with medium to high median temperatures, medium to high levels of precipitation, and medium to high elevational ranges are home to more alien bird species (S5 Table). Nevertheless, these abiotic effects are outperformed by native species richness as a determinant of alien species richness in our models (Table 1).

Cross-validation, holding out individual biogeographic realms (see Methods), confirms that the primary predictors in our global models are robust to sub-sampling of the data: colonisation pressure or native species richness was the strongest effect in all of the realm models (S6 Table), supporting the general influence of both anthropogenic and environmental drivers of species establishment and spread. Conversely, cross-validation reveals that time since introduction only enters models for alien species richness for the Indo-Malayan and Palearctic realms. A range of additional environmental variables are also retained in the most likely realm-level models, but their effects are always weak relative to those of colonisation pressure and native species richness (S6 Table).

We repeated our analyses excluding colonisation pressure to test whether lack of knowledge of this variable results in different conclusions about the determinants of alien species richness. This analysis shows that the influences of time since introduction and distance to historic port on alien species richness strengthen substantially (S7 Table). The resulting minimum adequate model additionally includes a positive effect of annual precipitation on alien species richness, suggesting that alien bird species richness is higher in areas of medium to high annual precipitation, as also tends to be the case for native species richness [19]. However, there is no evidence for direct abiotic effects when colonisation pressure is included (Table 1, S5 and S6 Tables). The minimum adequate model excluding colonisation pressure is also a substantially worse fit to the data (ΔwAIC = 1390.3 relative to the best model with colonisation pressure).

## Discussion

Biological invasions by alien species are one of the primary ways in which human activities are causing global environmental change, providing a strong incentive to understand the invasion process. The annual rate of first records of alien species worldwide has increased more or less constantly over the last 200 y and now averages >1.5 new records per day [23]. Nevertheless, the extent to which global variation in the richness of alien species is entirely the result of variation in human activities, or whether it also includes the imprint of the same sorts of natural processes that determine the richness of native species, is impossible to distinguish in the absence of studies that incorporate the effect of where species have been introduced—i.e., colonisation pressure [9]. Our global database on alien birds has enabled us to show, first, what underlies patterns in the global distribution of colonisation pressure, and second, how incorporating this information into analyses of alien species richness allows the relative influence of anthropogenic and natural drivers to be identified.

The drivers and locations of alien bird introductions have changed over the last 500 y. Early introductions were primarily planned with the aim of establishing new populations of beneficial species, although many bird species were also introduced for purposes of ornamentation [15,16]. The identities, sources, and introduction locations of the species concerned reflect these motivations, and the fact that early translocations (1500–1903 AD) were largely a European, and especially a British, endeavour. However, changes in the attitudes and legislation in the countries responsible for historical introductions have led to a reduction in colonisation pressure in those locations [18,24].

Recent introductions are higher on average in countries with higher per capita GDP, which is related to a country’s volume of trade as well as the disposable income of its populace. The identities, sources, and recipient locations of the species in recent introductions identify their origins primarily in unplanned releases of species in the cage bird trade, notably many parrots, finches, and mynas (S3 Table). The global locations with high current colonisation pressure (Fig 2D) reflect the present-day geographic foci of the bird trade, itself associated with increasing affluence and disposable income worldwide [17,25], but especially in rapidly developing countries in the Near and Far East [26,27]. Bird keeping is a popular hobby and an expression of social status in many countries, driving a growing and lucrative pet trade, especially in Eastern Asia, the Near East, and parts of Europe (Fig 2D) [28]. For example, bird introductions to Spain and Portugal exhibited a major increase in the early 1980s coinciding with economic upsurges in these countries and increasing volumes of trade [29]. Nevertheless, the relationship between GDP and number of introductions in recent years is triangular in form (Fig 3D); high GDP does not necessarily lead to higher colonisation pressure. Countries bucking this trend mainly include those in continental Europe, and notably also New Zealand, reflecting the changes in attitudes and legislation discussed above.

The identities of bird species involved in introductions have changed markedly over time (S3 and S4 Tables) but remain predominantly Old World in origin (c.f. Fig 2A and 2C, S1 Fig). The native ranges of recently introduced birds (Fig 2C) overlap the species richness hotspots of the Himalayan arc, tropical southeast Asia, and east Africa [30], but the distribution of red in Fig 2C suggests an overrepresentation of birds from open land biomes and an underrepresentation of forest birds. This could be because tropical forest species tend to have smaller geographic ranges, while bird species with larger geographic ranges are more likely to be introduced [3]. The biogeographic realm with the highest native bird species richness is the Neotropics [30], but this region remains relatively underexploited as a source for alien birds. A relatively high proportion of narrow-ranged species combined with the region’s relative distance from the main cage bird markets of the Near and Far East may be responsible for this situation. However, that may change as the bird trade exhausts supplies from other parts of the world [28], especially as the New World is home to many brightly coloured birds, adept songsters, and parrots, that would likely be highly marketable species. New World species such as *Aratinga jandaya*, *Mimus polyglottos*, and *Tangara seledon* have already been recorded for sale in Taiwanese bird shops [31]. It may also change if the demand for cage birds in Neotropical regions continues to grow. Fifty-nine bird species have already been recorded as introduced in Brazil, primarily from other parts of the Neotropics [32].

The historical drivers of colonisation pressure largely determine modern variation in alien species richness (Table 1), reflecting the direct effects of human activities on species distributions. The best global model (Table 1) explains 98.6% of the variation in bird alien species richness, accurately predicting alien species richness to *c*.2 species per grid cell (S6 Table), with colonisation pressure having the strongest effect (Table 1, S5 Table). Holdout cross-validation shows that colonisation pressure also explains high variation in alien species richness when removing four out of six biogeographic realms (S6 Table). Because (as previously noted) ASR is equal to colonisation pressure minus the number of introduced species that fail to establish, but plus the number of alien species that spread in from other areas, alien species richness is expected to be a positive function of colonisation pressure. Nevertheless, this result is not a given: patterns of failure and spread could have acted to erase the initial spatial variation in human introductions. The clear signature of colonisation pressure is therefore a cause for concern given the negative impacts of some alien species [5,6,7] and the rate at which colonisation pressure is increasing (Fig 1) [23]. Alien species richness is also higher where bird species have had more time to establish and spread following introduction. The early trade in alien birds was conducted largely by sea, and so areas proximal to historical sources of imported birds have higher alien species richness as a result. These results reinforce the need for greater controls on species introductions to prevent invasions, such as the 1993 Biosecurity Act in New Zealand or the recent EU Regulation No 1143/2014 on Invasive Alien Species. Also of concern is that the effects of time since first introduction and distance from a historic port imply an invasion debt [24], as modern sources of alien species are yet to obscure the imprint of historical processes. Interestingly, however, these effects are less consistent within biogeographic realms (S6 Table), suggesting that their global effect may be largely due to the different temporal and spatial invasion histories of different realms (Fig 2B).

Nevertheless, alien species richness is not just a function of human activities but also shows the imprint of natural processes. Notably, we find a consistent and strong positive association between alien species richness and native species richness, both globally (Table 1) and across realms (S6 Table); this runs counter to hypotheses of biotic resistance whereby diverse native assemblages resist the spread of alien species, but rather confirms previous analyses at island and continental scales showing that “the rich get richer” [33,34]. Previous studies have not controlled for colonisation pressure, opening the possibility that this phenomenon was purely a consequence of where species were introduced. Our results confirm that this association is independent of colonisation pressure. Most introductions have occurred at mid-latitudes (Fig 2B and 2D), which tend to have intermediate levels of native species richness [30], yet within the regions to which alien bird species have been introduced or spread, areas richer in native bird species tend also to be richer in aliens. Positive richness associations at broad scales might imply that native and alien bird species are responding to similar environmental variables [33], but what these variables are remains an important and open question. We also cannot rule out the alternative explanation that native and alien species are responding to different but spatially coincident processes. For example, alien bird richness may reflect some unmeasured aspect of human behaviour while humans tend to live at higher densities in areas rich in native bird species [35]. Nevertheless, our results show that, as a predictor of alien species richness, native species richness outperforms a variety of abiotic factors known to relate to bird species richness, including temperature, precipitation, and elevation (Table 1, S5 and S6 Tables).

Repeating the analysis as if data on colonisation pressure were not available results in a final model that is a substantially poorer fit to the data, which identifies stronger effects of time since first introduction and distance to historic port, and has additional effects of precipitation on alien species richness (c.f. Table 1 and S7 Table). Because alien species richness is expected to be a positive function of colonisation pressure, analyses of alien species richness carried out without data on this will assign higher importance to variables that are surrogates for colonisation pressure and will also give higher weighting to variables that may influence the number of introduced species that fail to establish, as well as the number of alien species that spread in from other areas. Upweighted variables may also influence alien species richness, as is the case for time since first introduction and distance to historic port in our analyses, but might include variables that would not otherwise rate as important, as is the case for precipitation. This model does identify that human activities matter to global variation in alien species richness; nevertheless, we can be more specific that the key activity in this regard is colonisation pressure. While the distribution of alien species richness and, in particular, the dearth of alien bird species in the arid zones (Fig 4) would indeed seem to imply that abiotic factors may be an important influence on where alien species richness is high or low, there is no evidence for direct abiotic effects when colonisation pressure is included (Table 1, S5 and S6 Tables). Instead, at least for birds, there were fewer attempts to establish alien species in areas of low precipitation.

Abiotic factors determine the fundamental niche of a species, delineating the set of environmental factors that determine where a species can and cannot maintain a population. Therefore, in some cases, the physical environment will influence the success or failure of alien species introductions and whether or not alien species established elsewhere can spread into a region [36]. Nevertheless, there are many areas of the world where abiotic conditions are suitable for species but where they are not naturally found because of biotic resistance (e.g., the presence of competitors) or dispersal limitation. Human-mediated translocation overcomes the second of these constraints, while the positive relationship between alien and native species richness suggests that the first may not be as strong a bar as has sometimes been hypothesised (S1 Table). The relatively weak influence of abiotic variables in our analyses may be because the likelihood that an area is within the fundamental niche of an alien is higher when it is within the fundamental niches of more native species, and, hence, the influence of the environment is subsumed within the effect of native species richness. Either way, our results highlight the need for further investigation of how environmental suitability and native richness might interact to determine the potential of different areas to accept alien species.

Environmental factors are unlikely to have a major impact on colonisation pressure, which has been largely driven by human activities, but will affect the number of introduced species that fail to establish and the number of alien species that spread into a region having been introduced elsewhere. The failure of individual introductions has been shown to depend on the number of individuals introduced (propagule pressure), traits of the species involved, and characteristics of the environment where the introduction took place [12,37]. For birds, establishment failure decreases as propagule pressure increases up to *c*.50–100 individuals, beyond which it is more strongly influenced by environmental suitability [12]. Our analysis does not consider which species fail or spread, only the number, but propagule pressure may affect this if it is systematically lower in some regions. In fact, propagule pressure is likely to be a positive function of colonisation pressure, especially for unplanned introductions [9], which may influence the precise form of the relationships we observe but will not alter the fact that alien species richness is a positive function of colonisation pressure.

Previous global studies have identified the donor and recipient regions of alien plants and regions with high alien plant richness [38] and shown that the distributions of gastropods after human transport are primarily explained by the prevailing climate and, to a smaller extent, by distance and trade relationships [39]. However, these studies have not had access to information on colonisation pressure, which we have shown to display interesting and informative spatial and temporal variation, and to be the key determinant of variation in the richness of alien bird species. Moreover, without information on colonisation pressure, statistical models are substantially poorer fits to data on alien species richness and differ in the factors they identify as richness drivers and the weights they assign to factors. Notably, alien species richness is not simply a consequence of higher overall levels of human activity (higher population, greater habitat disturbance, and increased access) in some regions, as it is independent of human footprint index, which quantifies this (S1 Table). Information on colonisation pressure is rarely available, but without it, erroneous conclusions regarding the determinants of alien species richness are likely. Including colonisation pressure allows us to understand, first, how changing histories of socioeconomic drivers affect the distribution and extent of bird population introductions (Fig 2), and then that alien species richness is a consequence of a combination of anthropogenic factors and biotic acceptance of aliens into areas already rich in native bird species.

## Methods

### Overview

Our analysis focuses on human-mediated introductions of bird species to locations outside their native geographic range. Our database (the Global Avian Invasions Atlas, or GAVIA; the acronym refers to the scientific name of the bird genus that includes divers or loons, although none of these species have ever been introduced) comprises 27,723 distribution records for 971 bird species for which there is some evidence of translocation outside their native range (i.e., aliens; see status categories below), based on almost 700 published references and substantial unpublished information derived from consultation with organisations and experts worldwide. Each entry in GAVIA corresponds to a single record of a single species recorded as introduced and alien in a specific location as published in a single reference. Records therefore correspond to descriptions or depictions of part or all of the alien geographic distribution of a species, rather than point occurrence records. For those records with a sufficient level of detail (for example, a sub-state or specific location of introduction was provided), or where the original reference contained a distribution map, vector range maps were created to represent the species’ alien ranges at different points in time. The full bird taxonomy used in GAVIA was that used by the International Union for the Conservation of Nature (IUCN) Red List of Threatened Species (www.iucnredlist.org, downloaded August 2010). The country and regional designations used in GAVIA were downloaded from the Global Administrative Areas (GADM) database (www.gadm.org, downloaded August 2010). GAVIA contains records for all statuses of introduction event, from those that were unsuccessful through to those that are deemed to be established, i.e., which are thought on the basis of population trends or expert local opinion to have a self-sustaining population in the area of introduction. More recent introductions are likely to be better catalogued, but the fact that bird distributions are very well known and that many of the historical introductions were planned and documented in detail [15,16] means that our data are likely to be of generally high quality [23]. The full GAVIA database is archived on Figshare at http://dx.doi.org/10.6084/m9.figshare.4234850 and described in Dyer et al. [40]; the specific data used in this paper are provided in supplementary files S1–S5 Data, as identified below.

Six categories were used in GAVIA to describe the invasive status of each alien species: (1) Established: The species has formed self-sustaining populations in the area of introduction; (2) Breeding: The species is known to be breeding/have bred in the area of introduction, but is not yet thought to be self-sustaining; (3) Unsuccessful: The species has not formed self-sustaining populations (casual, incidental); (4) Died Out: The species was once established but has now completely died out in the area of introduction; (5) Extirpated: The species was once established but has now been actively eradicated in the area of introduction; (6) Unknown: The status of the species in the area of introduction is not known and further clarification is necessary to determine which of the other five categories is appropriate. “Established species” refers to aliens in category 1 only, and “introduced species” to aliens in any category.

We use GAVIA to conduct two distinct analyses. First, we use data on the first introduction records of alien bird species between 1500 and 2000 AD to describe spatial and temporal variation in bird introductions worldwide. Specifically, we (i) divide records for the first appearance of each alien species in each country into quartiles based on record date. Focussing on the first (1500–1900 AD) and last (1983–2000 AD) quartiles, we then (ii) produce maps showing which countries alien birds had been introduced to and (iii) maps of the native distributions of those species. We present analyses to show (iv) whether colonial history or economic activity (GDP) can explain which countries received more alien bird introductions, (v) whether the number of countries receiving birds has increased over time, and (vi) whether the regions of origin of those bird species has changed over time. Finally, (vii) we test whether the identities of species introduced in the historical and modern eras are a random subset of all bird species. The methods for these analyses are presented in the next section (Spatial and Temporal Variation in Bird Introductions).

Second, we analyse global variation in the species richness of alien bird species at the 1° scale, incorporating information on colonisation pressure (first records arising from introduction rather than spread) as a key predictor of this variation. Specifically, we (viii) use data on the alien distributions of bird species to construct a global map of bird alien species richness at the scale of 1° grid cells and (ix) identify a set of anthropogenic and environmental variables that have been predicted to determine variation in alien species richness. We first (x) test for collinearity and (xi) spatial autocorrelation amongst the variables and use the outcomes of these tests to influence our final choices of predictor variables and methods. We then used (xii) Bayesian additive regression models and (xiii) simultaneous autoregressions to relate the chosen predictor variables to alien species richness while addressing the problem of spatial autocorrelation. We (xiv) use model selection approaches based on Akaike’s information criterion to identify key determinants of alien species richness and (xv) test the robustness of the predictors using holdout cross validation. The methods for these analyses are presented in the section entitled “Alien Bird Species Richness” below. All analyses were conducted using R (version 3.2.1) [41].

### Spatial and Temporal Variation in Bird Introductions

The subset of introduction records used here are those that represent the first record of a species in each country unit (countries, or states/provinces for Australia, Canada, and the United States) for the period 1500–2000 AD with known outcomes (statuses 1–5 above). Each species is therefore only counted in each country unit once, regardless of whether that introduction was successful or not. We censored introduction data to those falling within this time period because 1500 is a standard cutoff point for studying biological invasions [13,14], and there is evidence that introductions occurring after the year 2000 have not yet entered the literature and therefore represent an incomplete sample [23]. A very low proportion of recorded bird introductions are dated before 1500 AD (*c*.0.2%). We excluded from our analyses natural colonisations and translocations for conservation purposes. These criteria resulted in a total of 3,661 alien bird introduction records (first known occurrence of a given species in a given country unit) from 715 bird species (73.6% of species with introduced populations), which were distributed over time as shown in Fig 1. The data for Fig 1 are given in S1 Data.

(i) To facilitate comparison between historical and modern introductions, the 3,661 records were split into four quartiles on the basis of introduction date. The first quartile includes 922 records encompassing the period 1500–1903 AD, the second quartile 895 records from 1904–1956 AD, the third quartile 909 records from 1957–1982 AD, and the fourth quartile 935 introductions during the period 1983–2000 AD. Here, we present analyses on the first (“historical”) and fourth (“modern”) quartiles; the second and third quartiles are intermediate in their characteristics to these (c.f. Fig 2 and S1 Fig).

(ii) We constructed maps showing the countries into which species had been introduced in the historical and modern periods (Fig 2B and 2D) using introduction records from the GAVIA database, overlaid on the current map of countries (or country units) to avoid issues arising from historical changes in country boundaries. These maps show country-level estimates of colonisation pressure for the periods 1500–1903 AD (Fig 2B) and 1983–2000 AD (Fig 2D).

(iii) We constructed richness maps for the native distributions of these introduced species (Fig 2A and 2C) using native geographic range maps extracted from the database used by Orme et al. [30]; they were created by projecting the range maps onto a hexagonal grid of the world, resulting in a geodesic discrete global grid, defined on an icosahedron and projected onto the sphere using the inverse Icosahedral Snyder Equal Area projection. This resulted in a hexagonal grid composed of cells that retain their shape and area throughout the globe. These maps were created using ESRI ArcGIS version 10.2.2 [42].

(iv) To assess the influence of British colonial history on alien bird introductions (Fig 3), we obtained a list of former British colonies [43]. The countries in which alien species were recorded for the first time during the historical and modern periods of introduction were assigned to either “British colony” or “non-British colony” categories. A two-sample Wilcoxon test was used to compare the number of alien bird introductions in the historical time period (first quartile of introduction dates) in former British colonies, versus the number of introductions in non-British colonies. This was repeated for introductions in the modern era (fourth quartile) to determine whether the influence changed over the historical span of bird introductions.

To assess the influence of economic growth on alien bird introductions (Fig 3), data on GDP per capita (in 1990 international Geary-Khamis dollars [Int$]) were downloaded from ourworldindata.org (downloaded 18/03/15) [44]. We used per capita GDP because data are available for historical and modern time periods, it relates to the income of the populace, and countries with higher per-capita GDP tend also to have higher volumes of trade (r = 0.18, N = 207, *p* = 0.0066, for country-level GDP data from the UN and trade data from the CIA World Factbook, both sourced from Wikipedia on 25/08/16). Both income and trade are known to influence alien invasion pressure [17,26]. A subset of GDP data for the year 1900 was used for the countries present in the historical era, and the year 2000 for countries present in the modern era. GDP data were not available for all countries, particularly during the historical era, and countries without data were excluded, leaving 32 countries in the historical era analysis and 118 in the modern era. For the historical era, GDP per capita ranged from a minimum of Int$545 for China to a maximum of Int$5,899 for Switzerland (mean = Int$2,276; median = Int$1,980). For the modern era, GDP per capita ranged from a minimum of Int$509 for Sierra Leone to a maximum of Int$28,702 for the United States (mean = Int$7,162; median = Int$4,564). A linear regression was used to compare the number of alien bird introductions in the historical era and GDP per capita in the year 1900 (Int$). This was repeated for the modern era and GDP per capita in the year 2000 (Int$).

(v) Bespoke simulations were used to test for differences between the observed and expected number of country units where alien species had been first recorded in either time period. Each iteration of the simulation involved selecting 922 introductions at random, and without replacement, for the historical era (and 935 for the modern era) from the full dataset of all introductions in the period 1500–2000 (n = 3,661), and calculating the number of country units to which those introductions in this randomly chosen subset were assigned. This process was repeated 10,000 times for each time period, and the observed number of country units was judged significantly different from the expected if the observed number fell outside of the 2.5%–97.5% quantiles. Additionally, we calculated the number of overlapping countries between the historical and modern eras, for each of the iterations paired by iteration number (1–10,000), in order to determine if species were being introduced into the same or different countries in the different time periods. The observed overlap was judged to be significantly different to the expected if the observed number fell outside of the 2.5%–97.5% quantiles. The same procedure was also used to test for differences between the observed and expected number of species introduced in each time period.

(vi) In order to examine how the source locations of species introduced have changed between the two time periods (S4 Table), the native range of each species was intersected with the eight biogeographic realms defined by Olson et al. [45], using ESRI ArcGIS version 10.2.2 [42], and each was assigned to the realm where the native range of that species was found. No species from the Antarctic realm are included in these data, leaving seven realms in the analysis. For those species ranges that spanned more than one realm, the realm in which the largest part of the range fell was selected. Where the range was distributed equally across two or more realms, the species was excluded from the analysis (n = 27). This resulted in 225 species with assigned native realms in the historical era, and 298 species in the modern era (S4 Table). A Pearson’s Chi-squared test with a simulated *p*-value (based on 2,000 replicates) was used to determine whether the number of species sourced from each biogeographic realm was significantly different from that expected by chance between the historical era and the modern era.

(vii) Bespoke simulations were also used to test for differences between the observed and expected number of introduced species from each bird family in both time periods (S3 Table). For these randomisations, a list of the total global avifauna was used (n = 10,245 species [46]). Each iteration of the simulation involved selecting 245 species at random, and without replacement, for the historical era (324 for the modern era) from the total global avifauna and summing the number of these randomly chosen species in each family. Larger numbers of species are expected to be selected by chance from more speciose families. A total of 10,000 iterations of the simulation procedure were run for each time period, and the observed number of introduced species in any given family was judged significantly greater than the expected number if at least S% of the randomly derived values for that family were less than the observed, where S = (β/ 2) x 100. The β is calculated by applying a sequential Bonferroni correction to α, and α = 0.05 [47].

Lists of the numbers of introductions by country for the quartiles of introductions, as ranked by date, are provided in S2 Data, along with lists of introduced bird species for the first and fourth quartiles. Data on GDP and number introductions by country for quartiles 1 and 4 are provided in S3 Data. The data used to plot Fig 2 and S1 Fig are given in S4 Data.

### Alien Bird Species Richness

(viii) Global analyses of ASR were based on the vector range maps and introduction records from the GAVIA database, and additional raster data on environmental and anthropogenic variables. For consistency with studies of native bird species richness patterns [19,30], all data were converted to a global grid using a Behrmann equal area projection at a cell resolution of 96.486 km, equivalent to 1° longitude and approximately 1° latitude at the equator. This was performed using the *R* packages *sp* [48,49] and *raster* [50]. The global grid contained 360 by 152 cells, omitting the partial cells at latitudes higher than 87.13°. Each grid cell was assigned latitude and longitude values, which represented the centre point of each cell.

Alien species richness here is based on the 362 bird species with records of established (as defined above) alien populations (i.e., only those records in GAVIA with invasive status category 1) containing sufficient detail to convert to range maps using the software ESRI ArcGIS version 9.3 [51]. The most recently reported established range for each species was used to calculate alien species richness. The range maps were converted to grid cell counts using the *R* packages *rgdal* [52], *sp* [48,49], and *raster* [50]. Species were scored as present in a grid cell if any of the established introduced range fell within the cell boundaries. This ensured that even small established introductions, or those occurring on small islands, were counted. Overall alien species richness was derived by summing all species present within each cell and was mapped using ESRI ArcGIS version 10.2.2 (Fig 4) [42]. Alien species richness was natural log+1 transformed for analysis.

Overall, the global grid contained 54,720 cells, but cells not containing any alien bird species records would inflate covariation measures (the double zero problem [53]), and therefore those with either no alien bird introductions or to where no alien bird species had subsequently spread (i.e., where both colonisation pressure = 0 and ASR = 0) were excluded. Any cell with missing data for any of the variables described below was also excluded from the analysis, leaving a total of 10,258 grid cells.

### Predictor Variables

(ix) A set of anthropogenic and environmental predictor variables were selected for use in model building based on their suitability for hypothesis testing (S1 Table). Available raw data for each of the candidate variables were re-projected and re-sampled to the same equal area grid as the alien species richness data using spatial tools from R (for details see S2 Table) [41].

### Anthropogenic Variables

#### Colonisation pressure

Colonisation pressure (total number of species introduced to a grid cell) was calculated in a similar way to alien species richness, but included only the records from GAVIA which were considered “true” introductions, i.e., the locations where the species was considered to have escaped or been released, as opposed to subsequently spread. All records from GAVIA with invasive status categories 1–6 were considered for this variable. Only records for the 712 introduced species with sufficient detail on their colonisation pressure to enable conversion of their locations of introduction to range maps using the software ESRI ArcGIS version 9.3 [51] were included. The range maps were converted to grid cell counts using the *R* packages *rgdal* [52], *sp* [48,49], *raster* [50], and *rgeos* [54]. Species were scored as present in a grid cell if any of the introduced range fell within the cell boundaries, and colonisation pressure was calculated as the sum of all species introduced within each cell. Colonisation pressure was natural log+1 transformed for analysis.

#### Time since first introduction

Alien distribution records in GAVIA include a date of introduction, which is the first year that the species was recorded as being present in an area. To calculate the length of time since the first alien bird species was recorded in each grid cell, all of the dates of introduction for each species range map that overlapped each grid cell were extracted using the *R* packages *rgdal* [52], *sp* [48,49], and *raster* [50], and then the earliest date recorded from each cell was subtracted from the year 2015. This resulted in a single figure for each grid cell, equal to the number of years since the first record of any alien bird species in that cell. Time since first introduction was natural logarithmically transformed for analysis.

#### Human population density

The Gridded Population of the World: Population Density Grids gives data on global human population densities in 2000, adjusted to match UN totals, measured in persons per square kilometre. It was downloaded from http://sedac.ciesin.columbia.edu/gpw (Version 3 [GPWv3] [55], downloaded 26/04/2013). The *aggregate* function in *R* was used to extract the mean and median human population density for each grid cell. Human population density was natural log+1 transformed for analysis.

#### Human footprint index

The Global Human Footprint Dataset of the Last of the Wild Project is the Human Influence Index (HII) normalised by biome and realm. It was downloaded from http://dx.doi.org/10.7927/H4M61H5F (version 2 [55], downloaded 26/04/2013). The HII is a global dataset of 1-km grid cells, created from nine global data layers covering human population pressure (population density), human land use and infrastructure (land use/land cover, built-up areas, nighttime lights), and human access (roads, railroads, coastlines, navigable rivers). The *aggregate* function in *R* was used to extract the median human footprint index for each grid cell.

#### Distance to nearest city

Estimated travel time to the nearest city (>50,000 people in the year 2000) was downloaded from http://forobs.jrc.ec.europa.eu/products/gam/download.php [56] (accessed 10/09/2013) at a resolution of 30 arc seconds measured in minutes of travel time. It represents the travel time to the nearest city using land (road/off road) or water (navigable river, lake, and ocean) based travel. Cells containing a city were given a distance of zero. The *aggregate* function in *R* was used to extract the median travel time to the nearest city for each grid cell. Distance to nearest city was natural logarithmically transformed for analysis.

#### Distance to historic port

Distance to historic shipping port was based on the Climatological Database for the World’s Oceans: 1750–1854 (CLIWOC) dataset and was downloaded from http://pendientedemigracion.ucm.es/info/cliwoc/cliwoc15.htm (accessed 08/04/2015) [57]. The *dist* function in *R* was used to find the distance in metres from the centre of each grid cell to the nearest port in the database. Ports were identified as grid cells containing boats that were recorded as "anchored." Distance to historic port was divided by 1,000 to convert it to kilometres and then square-root transformed for analysis.

### Environmental Variables

#### Native bird species richness

Native bird species richness for 9,650 extant bird species was calculated using native breeding range data obtained from the ADHoC (Avian Diversity Hotspots Consortium) database, first published by Orme et al. [30]. Native bird species richness was calculated using the *R* packages *rgdal* [52], *sp* [48,49], and *raster* [50], and species were scored as present in a grid cell if any of the native range fell within the cell boundaries. Native bird species richness was square-root transformed for analysis.

#### Elevation (median and range)

Elevational data were downloaded from http://www.worldclim.org/ [58] (downloaded 01/05/2013) at a resolution of 30 arc seconds measured in metres above sea level. The *aggregate* function in *R* was used to extract the minimum, maximum, and median elevations for the land area within each grid cell. The minimum was subtracted from the maximum to obtain the elevational range of each cell. In 44 of the grid cells, median elevation was below sea level, and therefore had a negative value (0.43% of all cells). The most negative median elevation value was -29.7; therefore, 30 was added to all median elevation values before they were logarithmically transformed. Elevational range was square root transformed for analysis.

#### Temperature (median, minimum, maximum and range)

Data on temperature were downloaded from http://www.worldclim.org/ [58] (downloaded 01/05/2013) at a resolution of 30 arc seconds measured in degrees centigrade multiplied by ten. The *aggregate* function in *R* was used to extract the median temperature within each cell from the WorldClim bioclimatic variable BIO1 (annual mean temperature), the minimum from bioclimatic variable BIO6 (minimum temperature of coldest month), and the maximum from bioclimatic variable BIO5 (maximum temperature of warmest month). The minimum, maximum, and median temperatures were divided by ten in order to convert them back to true centigrade values. The minimum was then subtracted from the maximum in order to obtain the temperature range within each grid cell. Temperature range was square root transformed for analysis.

#### Precipitation (median)

Data on precipitation were downloaded from http://www.worldclim.org/ [58] (downloaded 01/05/2013) at a resolution of 30 arc seconds measured in millimetres. The *aggregate* function in *R* was used to calculate the median precipitation within each cell from the WorldClim bioclimatic variable BIO12 (annual precipitation). Precipitation was square root transformed for analysis.

#### Habitat complexity

Data on land cover types were downloaded from http://www.esa-landcover-cci.org/?q=node/158 [59] (downloaded 02/04/2015) and included all 37 categories at a resolution of 300 m. The *extract* function from the *R* package *raster* [50] was used to extract the number of different land cover types in the surrounding 8 and 24 cells of each grid cell as a measure of habitat complexity.

### Statistical Analyses

(x) Tests of collinearity between the predictor variables found relatively high correlations within the temperature variables, elevation variables, and habitat complexity variables, and between human population density, human footprint index, and distance to the nearest city (S8 Table). The predictor variables temperature minimum, maximum, and range, median elevation, and the habitat complexity in surrounding 24 cells were thus excluded from models *a priori*. As the human footprint index incorporated human population density, human infrastructure, and road access, population density mean and median and distance to city were also excluded *a priori*. This resulted in nine predictor variables for analysis.

### Spatial Autocorrelation

(xi) Spatial autocorrelation is a common phenomenon in environmental data, where similarities in the values of predictor and response variables arise as a function of proximity of sampling locations. Species distribution data in particular are inherently spatially structured [60] due to a combination of intrinsic processes such as population growth and dispersal [61], areas of false presence or absence records due to errors in distributional data [62], or where the environmental processes that drive species richness patterns show spatial autocorrelation themselves [61]. There is strong spatial autocorrelation in both the response and predictor variables in our data (Moran’s I ≥ 0.74; *p* < 0.001 in all cases, with the exception of habitat complexity, for which Moran’s I = 0.21; *p* < 0.001), and therefore regression methods that assume each grid cell is an independent data point are inappropriate here.

### Statistical Approaches

Given the problem of spatial autocorrelation, we analysed variation in alien species richness in two alternate ways.

(xii) First, we conducted the analysis using a spatially structured random effect. We used stochastic partial differential equations (SPDE) [63] to fit a Gaussian random field to the data to approximate the patterns of spatial autocorrelation. We included this effect in a Bayesian additive regression model inferred using integrated Laplace approximations (INLA) in R (R-INLA) [64]. We tested several different versions of the spatial mesh, choosing complexity using Watanabe-AIC (wAIC) [65] to estimate the benefit of increasing the complexity of the spatial term versus its ability to explain complex spatial patterns. As the dependent variable (alien species richness) had a right-skewed distribution, we tested several candidate error “families” (Gaussian, log-Gaussian, Poisson, negative binomial) and compared these models using the conditional predictive ordinate (CPO) measure of fit, which gives the probability of each individual data point given the model (and ranges from 0 in the case of poor fit, to N [the sample size] in the case of perfect fit), and the probability integral transform (PIT) values. The log-Gaussian distribution was the best fit to the data with zero CPO failures, a right-skewed CPO histogram, showing that very few values had a low probability given the data and a uniform PIT distribution [66].

(xiii) To validate the INLA results, we also analysed variation in alien species richness using simultaneous autoregressions (SAR). Neighbourhood size was defined as the distance that captured the centre point of all eight surrounding grid cells (150 km). Neighbourhood connection matrices were calculated with row-standardised weights. Two specifications of the error covariance matrix were considered: SAR_lag_ (spatial autocorrelation in the response) and SAR_err_ (spatial autocorrelation in the error term). A Lagrange multiplier test was used to find the best error specification, and the SAR_err_ model showed higher support (SAR_err_: Lagrange multiplier LMerr = 20,876, *p* < 0.001; SAR_lag_: Lagrange multiplier LMlag = 15,219, *p* < 0.001). SAR_err_ models are recommended as most reliable when dealing with spatially autocorrelated species distribution data, having been found to perform well and provide the most precise parameter estimates regardless of the kind of spatial autocorrelation and whether model selection is via *R*^2^ or AIC [60]. All SAR models were constructed with the *R* package *spdep* [67,68].

### Model Selection

(xiv) We ran single predictor models of all variables in order to compare the significance and directions of slopes for different predictors modelled in isolation (S5 Table). We included quadratic as well as linear terms for six of the predictors in our models to allow for non-linear relationships (colonisation pressure, time since introduction, native species richness, elevational range, median temperature, and precipitation). The inclusion of quadratic terms was determined by comparing single predictor models for each linear term with a model containing both the linear and quadratic form. For the SAR regression mode, if the AIC (Akaike’s information criterion) [69] improved by >4 then the quadratic form was included in model building. For the INLA model we used the analogous Watanabe-AIC (wAIC) [65], also with a threshold of 4.

A multivariate minimum adequate model (MAM) was generated by forward stepwise procedures. The single predictor model with the lowest AIC or wAIC value was used as a starting model, with each “next best” predictor added in turn. The criterion for inclusion of additional model terms was improvement of the AIC value by >4. The use of an AIC type approach in model selection procedures observes principles of parsimony and avoids the model over-fitting that can be a result of data dredging [69]. Once the MAM with the lowest overall AIC score was identified, each predictor not included was once again added in turn to ensure that the best combination of predictor variables was selected. For the INLA models, ∑CPO, another goodness-of-fit measure, was also calculated, where higher scores represent more data points having high probability given the model (S3 Fig). A maximum model fit for INLA or SAR models was also assessed with a pseudo-*R*^*2*^ value calculated as the squared Pearson correlation between predicted and observed values (S3 Fig) [60]. These values are for illustrative purposes only and should not be overinterpreted. For the INLA models, spatial plots of residuals show that autocorrelation is virtually eliminated by including a spatial term (S4 Fig). For SAR models, spatial correlograms were used to examine the patterns of spatial autocorrelation for alien bird species richness and for the model residuals, and confirm that the method also largely eliminates spatial autocorrelation in the MAM (S5 Fig).

(xv) To assess our model selection methods and the robustness of model parameter estimates, holdout cross validation was performed (S6 Table) in a manner akin to k-fold cross validation [70], but following Newbold et al. [71] using realms to test the biological predictions of the models. This allowed us to provide a more realistic biological test of model predictions, rather than using hold-out models on randomly divided sets of data. Each of the 10,258 grid cells was assigned to a biogeographic realm [45]. The grid cells from the Antarctic and Oceanic realms were excluded from this part of the analysis due to low sample size. Five of the six remaining realms were used as the training set upon which stepwise model selection was conducted, as described above. The sixth realm was then used as the testing set, and a cross validation metric, root mean squared error (RMSE), was calculated to assess the ability of the final model at predicting the held-out realm. This process was repeated five more times with a different realm being used as the testing set each time, and then the RMSE across all samples was averaged to obtain the mean cross validation error, which provides an estimate of the predictive accuracy of our full data model if challenged by new data.

INLA and SAR models gave qualitatively similar results, identifying the same key predictors of global spatial variation in alien species richness. Therefore, we focus on the results of the INLA analysis, as this was the more conservative of the two approaches. The equivalent SAR results are available on request. The data frame for the analysis of alien species richness is provided in S5 Data.

## Supporting Information

S1 Fig Global maps showing the richness of the native ranges of the alien bird species. Global maps showing the richness of the native ranges of the alien bird species introduced during (a) the second quartile (1904–1956AD) and (b) the third quartile (1957–1982AD) of the data (the first and fourth quartiles are shown in Fig 2A and 2C). Cold colours represent lower density of bird species, warm colours represent higher density. Grey areas are those not covered by the native ranges of the species.
(DOCX)

S2 Fig Univariate relationships to log (1 + Alien Species Richness). (a) log (1 + Colonisation Pressure), (b) log time since first introduction, (c) sqrt distance to historic port, and (d) sqrt native species richness. The coefficients for the relationships are given in S5 Table, and further details of the variables in the Methods.
(TIF)

S3 Fig Diagnostic plots for INLA regression. (a) Histogram of the posterior means of the predictive distribution, with low number of low and high probabilities, (b) regression of observed on fitted values, showing the strong fit between the two.
(TIF)

S4 Fig Spatial patterns of residuals from an INLA regression. (a) Residuals with full covariates but without a spatial term; (b) Residuals with a spatially structured random effect. Redder colours indicate more positive residuals and bluer more negative, with yellow closer to zero.
(TIF)

S5 Fig Correlograms to examine the patterns of spatial autocorrelation. Correlograms concern (a) alien bird richness; (b) the residuals of the most likely SARerr model.
(DOCX)

S1 Table Predicted relationships between alien bird species richness and anthropogenic and environmental variables. The predicted relationship and studies that provide support for each prediction are given.
(DOCX)

S2 Table Details of the anthropogenic and environmental predictor variables selected for use in model building. (DOCX)

S3 Table The distribution of introduced birds by family. The number of species introduced from a family in the first quartile (historical) and fourth quartile (modern), the total number of species in the family (Total) [46], and the probability (Psim calculated using simulations; see Methods) of observing as many or more introductions (or fewer introductions for those in the shaded areas) from that family given the number of species in the family and the proportion of the world’s bird species that have been introduced. The families shown are those with probabilities that are significantly lower than expected (α = 0.05), once a sequential Bonferroni correction for multiple statistical tests [72] has been applied.
(DOCX)

S4 Table The number of species from different biogeographic regions that have been introduced outside of their native range in the historic and modern eras. Prop. historic = the proportion of species introduced in the first quartile of bird introductions (historical era) from each biogeographic region; Prop. modern = the proportion of species introduced in in the fourth quartile of bird introductions (modern era) from each biogeographic region. The numbers (and proportions) of species sourced from each biogeographic realm during the two time periods differed significantly (chi^2^ = 22.88, p <0.001).
(DOCX)

S5 Table Outputs from single predictor INLA models where log+1 alien species richness is the response variable. S.E. = standard error for the variable. ∑CPO = the sum of the probabilities of each data point given the model. For comparison, fitting an intercept only model gives wAIC = –11,610.6 and CPO = 4,457.5.
(DOCX)

S6 Table Impact of predictor variables included in each of the holdout cross validation INLA models measured in AIC units. The shaded column indicates the selected predictors and values for the minimum adequate model using all of the data. Goodness-of-fit was calculated with Pearson’s correlation coefficients between the response variables and the fitted values of the models (i.e. pseudo R^2^). Average RMSE of holdout models = 0.735 (i.e. overall predictive accuracy = e^0.725^ = 2.08 species per grid cell). MAM = minimum adequate model. RMSE = root mean squared error. AIC = Akaike’s Information Criterion.
(DOCX)

S7 Table Spatial correlates of alien bird richness for the minimum adequate model excluding colonisation pressure. Parameter estimates are given fitting a Gaussian random field to the data to approximate the patterns of spatial autocorrelation in a Bayesian additive regression model inferred using INLA. wAIC = –12,449, conditional predictive ordinate (CPO) measure of fit = 4,797.2. For comparison, fitting an intercept only model gives wAIC = –11,610.6 and CPO = 4,457.5. S.E. = standard error. n = 10,258 grid cells.
(DOCX)

S8 Table Correlation matrix of all transformed predictor variables. r is above the diagonal and P is below it.
(DOCX)

S1 Data Data on number of introductions in different time periods used in Fig 1. (XLSX)

S2 Data The numbers of introductions by country for the first and fourth quartiles of introductions, as ranked by date, and lists of the introduced bird species for these quartiles. (XLSX)

S3 Data Data on GDP and number introductions by country for the first and fourth quartiles of introductions, as ranked by date. (XLSX)

S4 Data Hexagonal grid cell data used to plot Fig 2 and S1 Fig. (XLSX)

S5 Data The data frame for the analysis of alien species richness. (XLSX)

## Abbreviations

ASR: alien species richness
GADM: Global Administrative Areas
GAVIA: Global Avian Invasions Atlas
IUCN: International Union for the Conservation of Nature
NSR: native species richness
