# Supplementary figures and images for: The Global Distribution and Drivers of Alien Bird Species Richness
Source: PLoS Biol. 2017 Jan 12;15(1):e2000942. doi: 10.1371/journal.pbio.2000942 (PMC5230740; doi:10.1371/journal.pbio.2000942)

a)


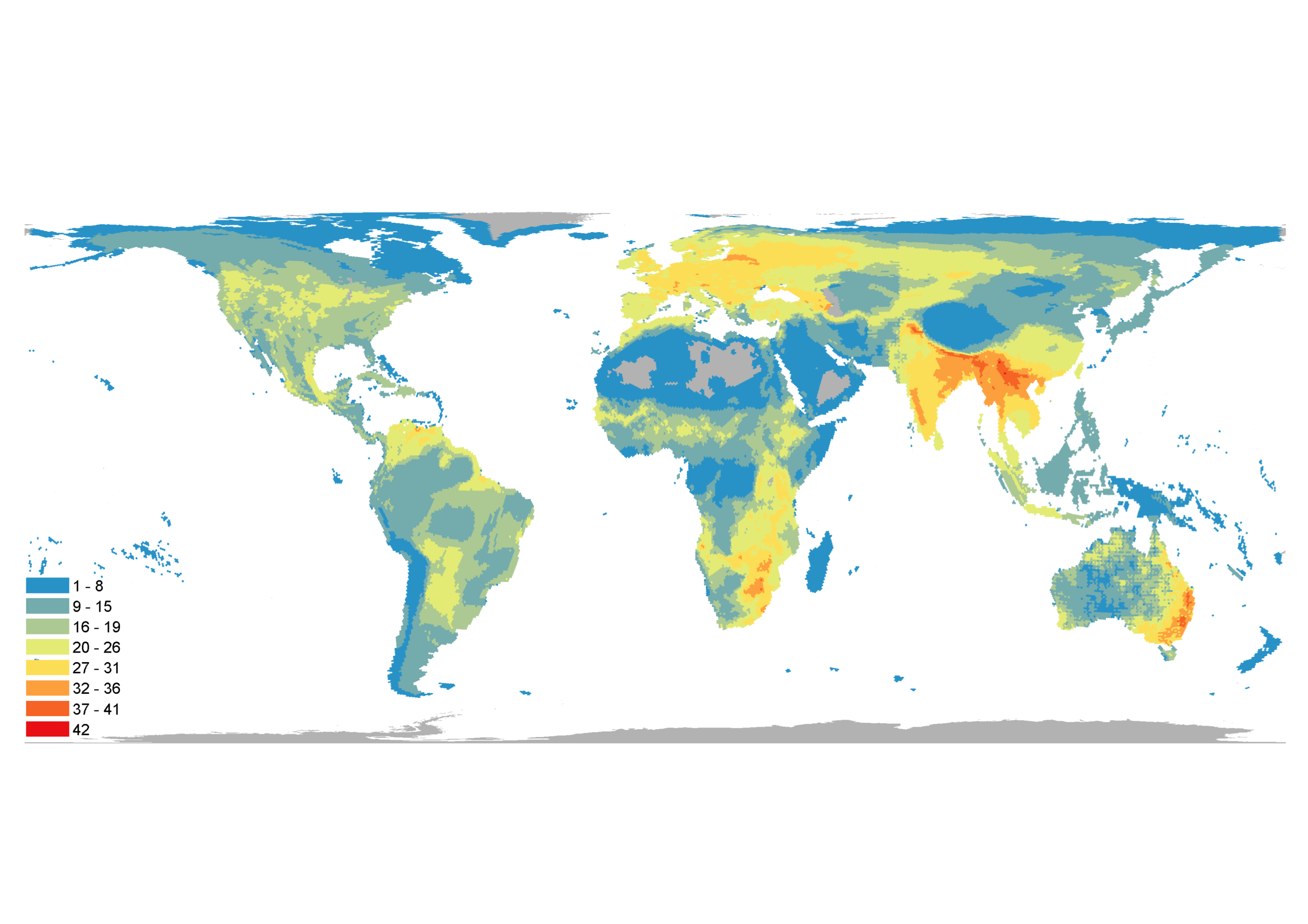
b)


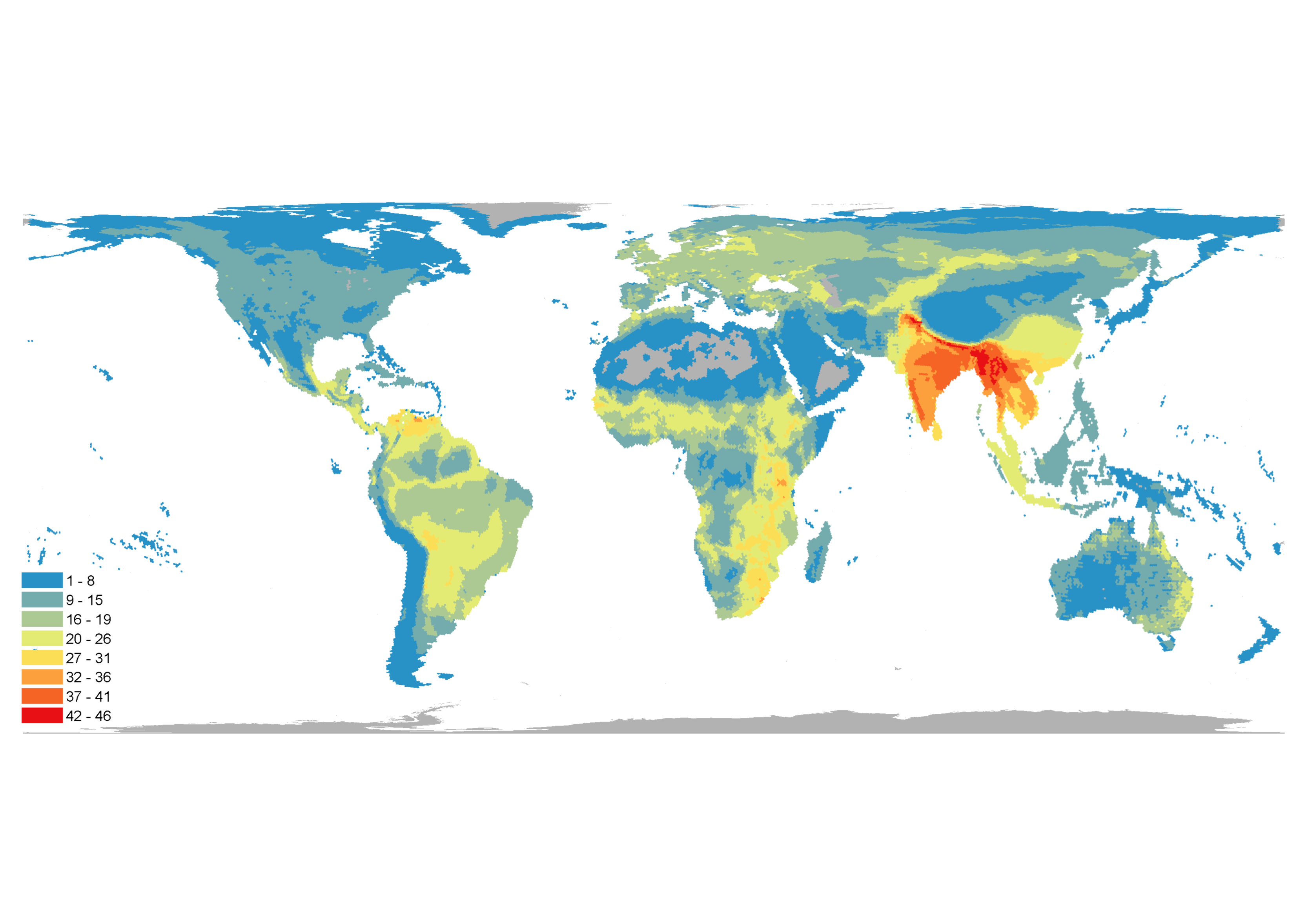

Supplement: S1 Fig — Global maps showing the richness of the native ranges of the alien bird species introduced during (a) the second quartile (1904–1956AD) and (b) the third quartile (1957–1982AD) of the data (the first and fourth quartiles are shown in Fig 2A and 2C). Cold colours represent lower density of bird species, warm colours represent higher density. Grey areas are those not covered by the native ranges of the species. (DOCX) [file pbio.2000942.s001.docx]

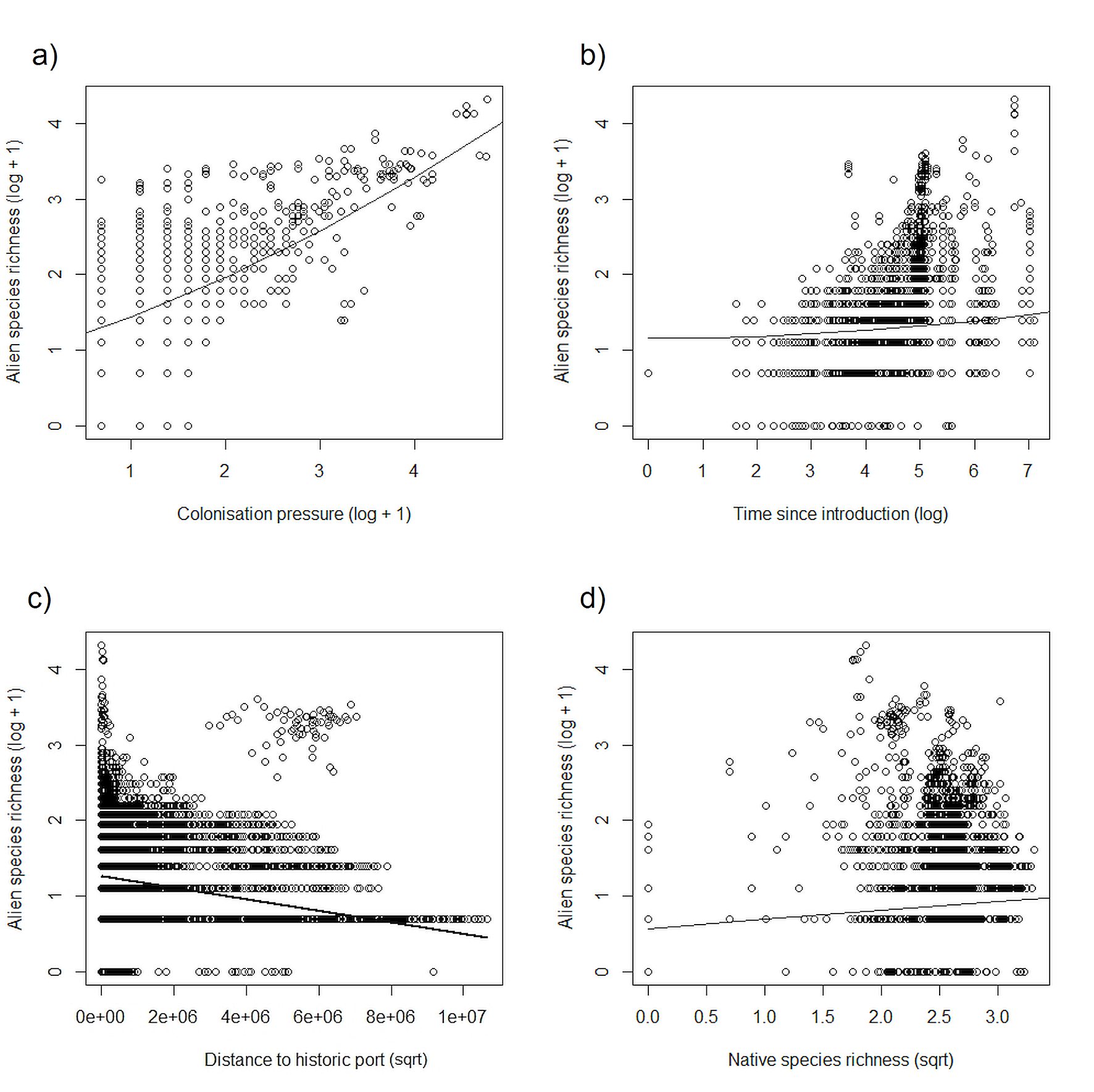

Supplement: S2 Fig — (a) log (1 + Colonisation Pressure), (b) log time since first introduction, (c) sqrt distance to historic port, and (d) sqrt native species richness. The coefficients for the relationships are given in S5 Table, and further details of the variables in the Methods. (TIF) [file pbio.2000942.s002.tif]

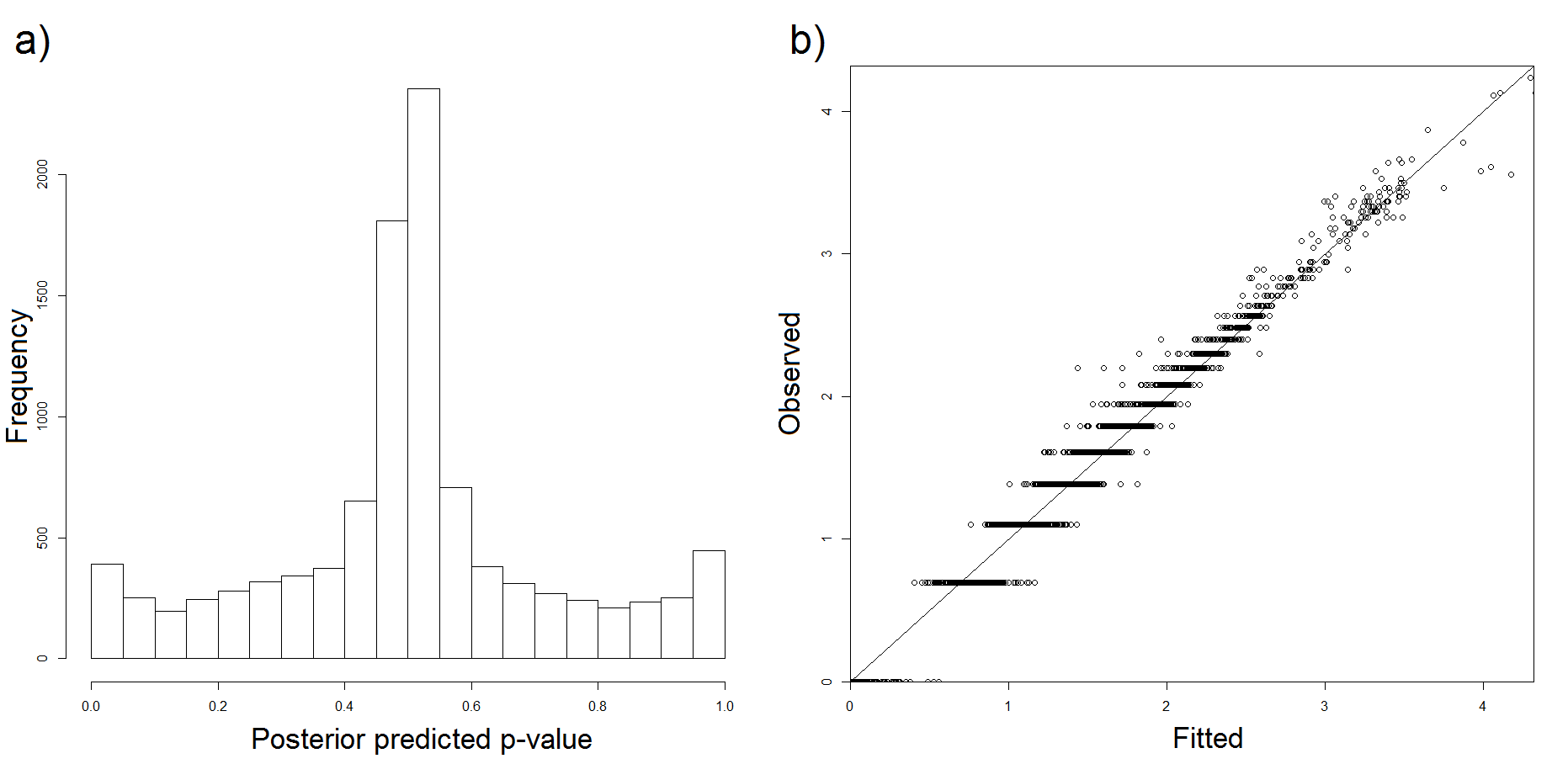

Supplement: S3 Fig — (a) Histogram of the posterior means of the predictive distribution, with low number of low and high probabilities, (b) regression of observed on fitted values, showing the strong fit between the two. (TIF) [file pbio.2000942.s003.tif]

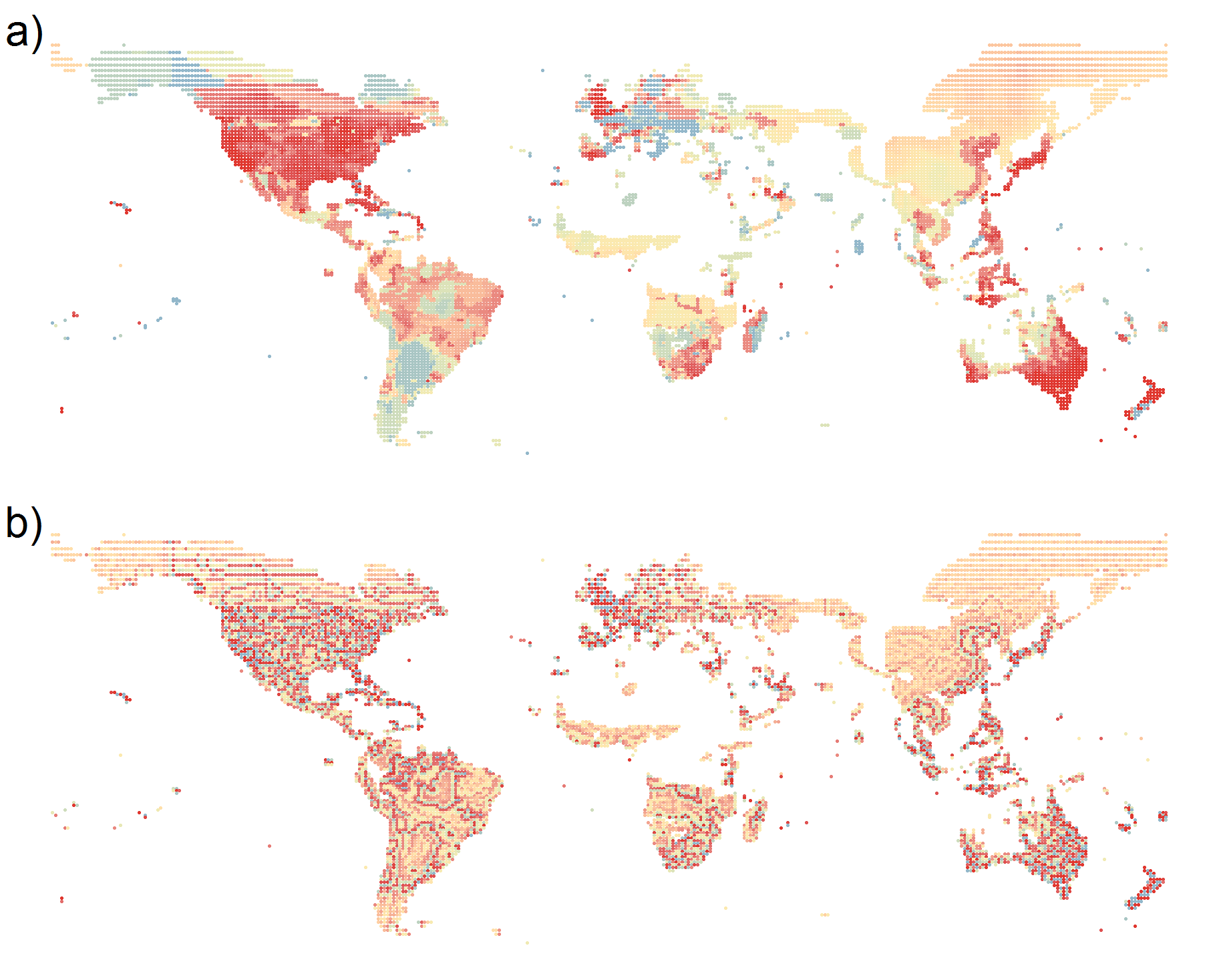

Supplement: S4 Fig — (a) Residuals with full covariates but without a spatial term; (b) Residuals with a spatially structured random effect. Redder colours indicate more positive residuals and bluer more negative, with yellow closer to zero. (TIF) [file pbio.2000942.s004.tif]

a)


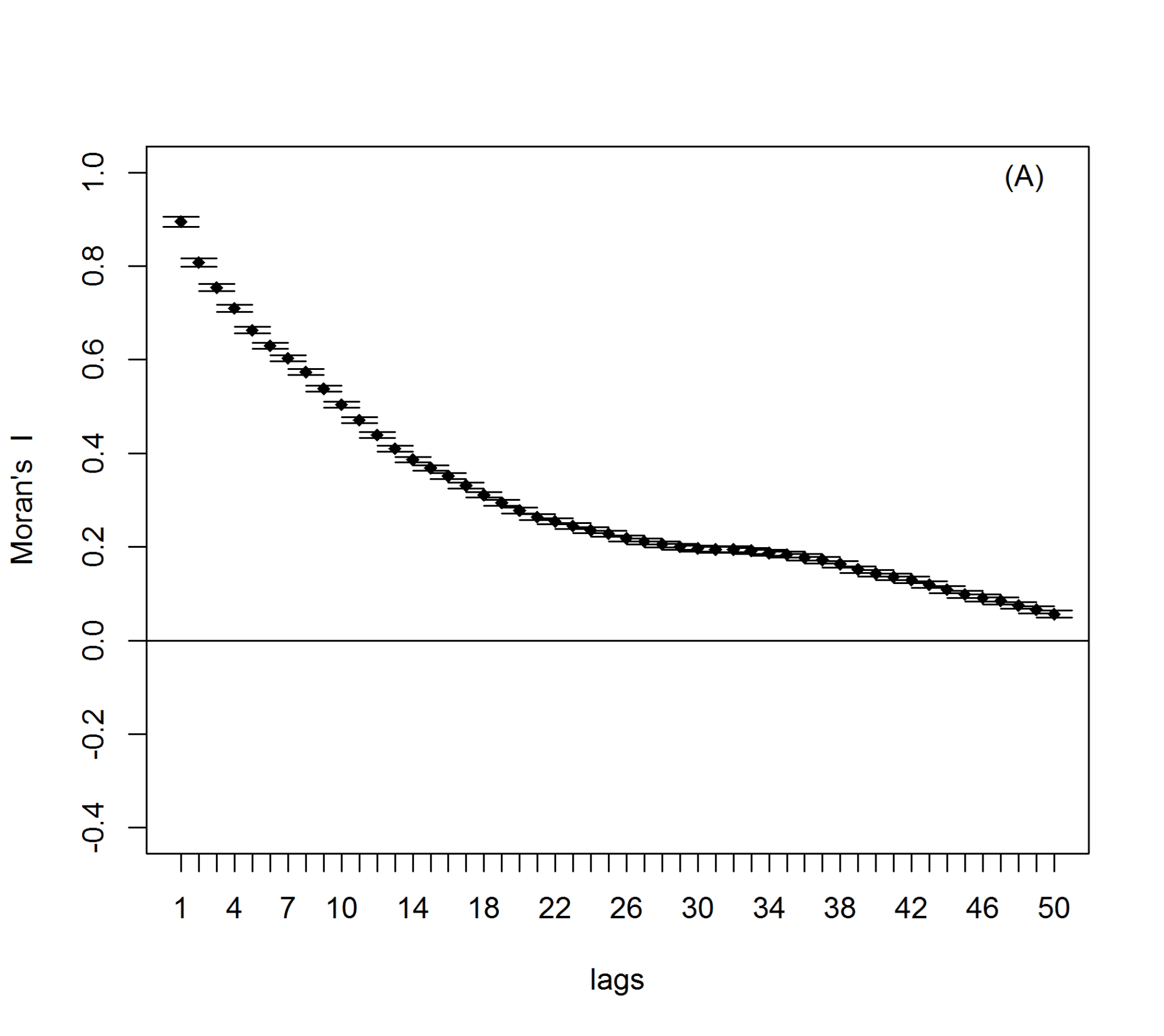


b)


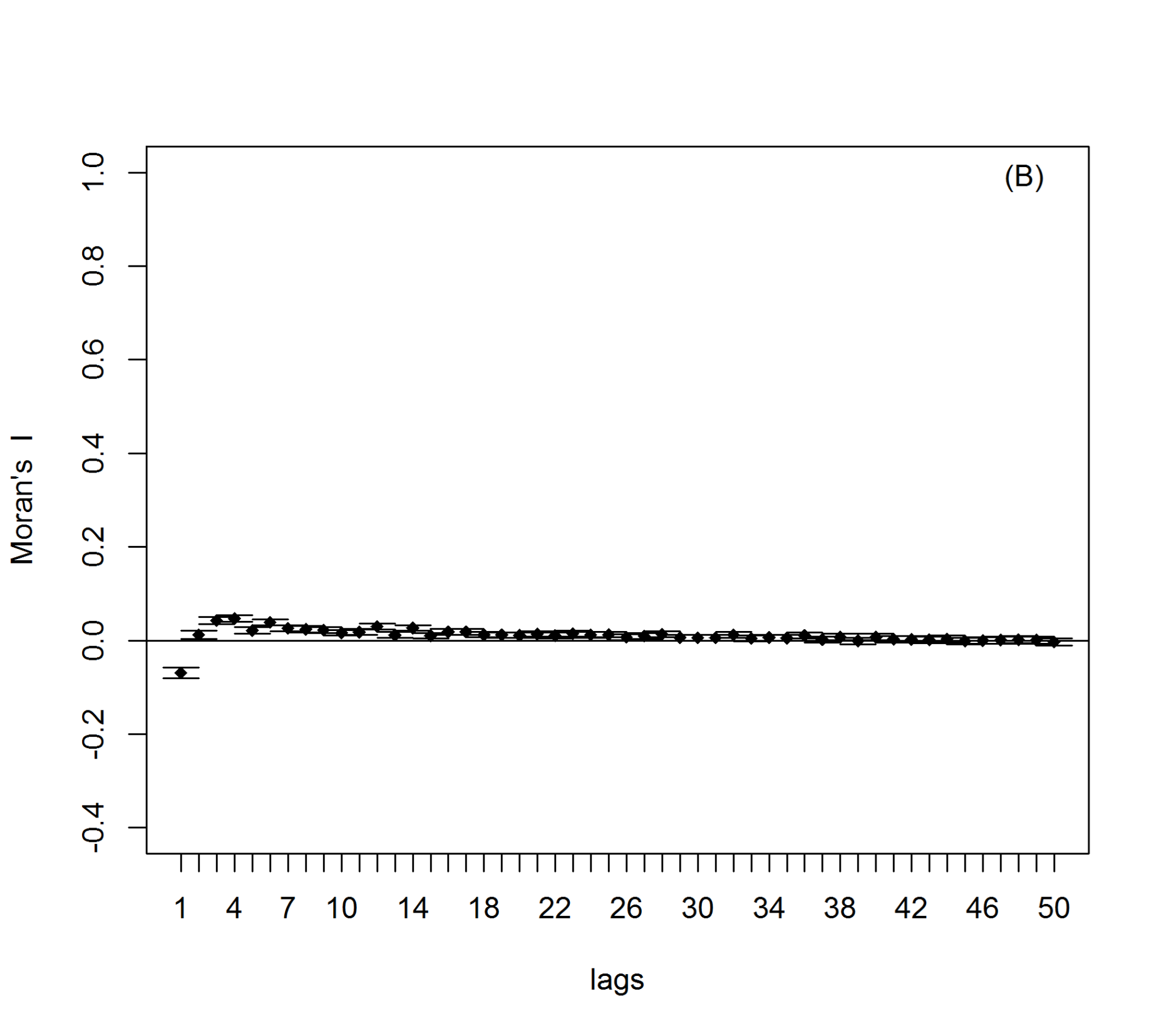

Supplement: S5 Fig — Correlograms concern (a) alien bird richness; (b) the residuals of the most likely SARerr model. (DOCX) [file pbio.2000942.s005.docx]
