# Supplementary material for: The Global Distribution and Drivers of Alien Bird Species Richness
Source: PLoS Biol. 2017 Jan 12;15(1):e2000942. doi: 10.1371/journal.pbio.2000942 (PMC5230740; doi:10.1371/journal.pbio.2000942)
Supplement: S1 Table — The predicted relationship and studies that provide support for each prediction are given. (DOCX) [file pbio.2000942.s006.docx]

|  |  | Prediction | Predicted relationship | Support |
| --- | --- | --- | --- | --- |
| **Anthropogenic** | |  |  |  |
|  | Colonisation pressure | As colonisation pressure (i.e. the number of species introduced) increases, so will alien bird richness | + | [1–4] |
|  | Time since first introduction | Species richness will be highest in areas where alien birds have been introduced for the longest | + | [5–7] |
|  | Human population density | Alien species richness will be highest in areas where human population density is high | + | [2,8–11] |
|  | Human footprint index | Where human footprint is high (including higher human population, greater habitat disturbance and increased access) alien bird richness will also be high | + | [10–14] |
|  | Distance to city | As you move further away from cities, alien bird richness will decrease | - | [15] |
|  | Distance to historic port | As you move further away from historic ports, alien bird richness will decrease | - | [9,11,12] |
|  |  |  |  |  |

| **Environmental** | |  |  | | |  | |
| --- | --- | --- | --- | --- | --- | --- | --- |
| ***Biotic*** | |  |  | | |  | |
|  | Native species richness | In areas where native bird richness is high, alien bird richness will also be high due to biotic acceptance | | + | [16] | |  |
|  |  | In areas where native bird richness is high, alien bird richness will be low due to biotic resistance | | ­- | [15] | |  |
|  | Habitat complexity | As the number of habitat types in an area increases, as will alien species richness | | + | [17,18] | |  |
| ***Abiotic*** |  |  | |  |  | |  |
|  | Elevation | As elevational range increases, alien bird richness will also increase | | + | [19–22] | |  |
|  | Temperature | Alien bird richness will be low at low temperatures, and higher at mid-to high temperatures. | | -/+ | [19,23,24] | |  |
|  | Precipitation | As the level of precipitation increases, so will alien species richness | | + | [25–27] | |  |

**References**

1. Jeschke JM, Strayer DL. Invasion success of vertebrates in Europe and North America. Proc Natl Acad Sci USA. 2005; 102: 7198–7202.

2. Blackburn TM, Lockwood JL, Cassey P. The island biogeography of exotic bird species. Glob Ecol Biogeogr. 2008; 17: 246–251.

3. Chiron F, Shirley SM, Kark S. Human-related processes drive the richness of exotic birds in Europe. Proc R Soc Lond B. 2009; 276: 47–53.

4. Van Wilgen NJ, Richardson DM. The roles of climate, phylogenetic relatedness, introduction effort, and reproductive traits in the establishment of non-native reptiles and amphibians. Conserv Biol. 2012; 26: 267–277.

5. Wilson JRU, Richardson DM, Rouget M, Proches S, Amis MA, Henderson LS, et al. Residence time and potential range: crucial considerations in modelling plant invasions. Divers Distrib. 2007;13: 11–22.

6. Li Y, Liu X, Li X, Petitpierre B, Guisan A. Residence time, expansion toward the equator in the invaded range and native range size matter to climatic niche shifts in non-native species. Glob Ecol Biogeogr. 2014; 23: 1094–1104.

7. Byers JE, Smith RS, Pringle JM, Clark GF, Gribben PE, Hewitt CL, et al. Invasion expansion: Time since introduction best predicts global ranges of marine invaders. Sci Rep. 2015; 5: 1–9.

8. Pyšek P, Jarošík V, Hulme PE, Kühn I, Wild J, Arianoutsou M, et al. Disentangling the role of environmental and human pressures on biological invasions across Europe. Proc Natl Acad Sci. 2010;107: 12157–12162.

9. Essl F, Dullinger S, Rabitsch W, Hulme PE, Hülber K, Jarosik V, et al. Socioeconomic legacy yields an invasion debt. Proc Natl Acad Sci USA. 2011;108: 203–207

10. Spear D, Foxcroft LC, Bezuidenhout H, McGeoch MA. Human population density explains alien species richness in protected areas. Biol Conserv. 2013; 159: 137–147.

11. Hulme PE. Trade, transport and trouble: managing invasive species pathways in an era of globalization. J Appl Ecol. 2009; 46: 10–18.

12. Gallardo B, Zieritz A, Aldridge DC. The importance of the human footprint in shaping the global distribution of terrestrial, freshwater and marine invaders. PLoS ONE. 2015; 10: 1–17.

13. Sharma GP, Esler KJ, Blignaut JN. Determining the relationship between invasive alien species density and a country’s socio-economic status. S Afr J Sci. 2010; 106: 1–6.

14. Westphal MI, Browne M, MacKinnon K, Noble I. The link between international trade and the global distribution of invasive alien species. Biol Inv. 2008; 10: 391–398.

15. Elton C. The ecology of invasions by animals and plants. Methuen; 1958.

16. Stohlgren TJ, Barnett D, Flather C, Fuller P, Peterjohn B, Kartesz J, et al. Species richness and patterns of invasion in plants, birds, and fishes in the United States. Biol Inv. 2006; 8: 443–463.

17. Rosenzweig ML. Species diversity in space and time. Cambridge: Cambridge University Press; 1995.

18. Kerr JT, Southwood TRE, Cihlar J. Remotely sensed habitat diversity predicts butterfly species richness and community similarity in Canada. Proc Natl Acad Sci USA. 2001; 98: 11365–11370.

19. Davies RG, Orme CDL, Storch D, Olson V, Thomas GH, Bennett PM, et al. Topography, temperature and the global distribution of bird species richness. Proc R Soc B-Biological Sci. 2007;274: 1189–1197.

20. Richerson PJ, Lum K. Patterns of plant species diversity in California: relation to weather and topography. Am Nat. 1980; 116: 504–536.

21. Kerr JT, Packer L. Habitat heterogeneity as a determinant of mammal species richness in high-energy regions. Nature. 1997; 385: 252–254.

22. Rahbek C, Graves GR. Multiscale assessment of patterns of avian species richness. Proc Natl Acad Sci USA. 2001; 98: 4534–4539.

23. Turner JRG, Lennon JJ, Lawrenson JA. British bird species distributions and energy theory. Nature. 1988; 335: 539–541.

24. Currie DJ. Energy and large-scale patterns of animal- and plant-species richness. Am Nat. 1991; 137: 27–49.

25. Orme CDL, Davies RG, Burgess M, Eigenbrod F, Pickup N, Olson VA, et al. Global hotspots of species richness are not congruent with endemism or threat. Nature. 2005; 436: 1016–1019.

26. Waide RB, Willig MR, Steiner CF, Mittelbach GC, Gough L, Dodson SI, et al. The relationship between net primary productivity and species richness. Annu Rev Ecol Syst. 1999; 30: 257–300.

27. Essl F, Dullinger S, Moser D, Steinbauer K, Mang, T. Macroecology of global bryophyte invasions at different invasion stages. Ecography. 2015; 38: 488–498.
