# Supplementary material for: The Global Distribution and Drivers of Alien Bird Species Richness
Source: PLoS Biol. 2017 Jan 12;15(1):e2000942. doi: 10.1371/journal.pbio.2000942 (PMC5230740; doi:10.1371/journal.pbio.2000942)
Supplement: S2 Table — (DOCX) [file pbio.2000942.s007.docx]

| **Predictor** | | **Variations (per grid cell)** | **Transformations** | **Raw resolution** | **Unit** | **Source** |
| --- | --- | --- | --- | --- | --- | --- |
| **Anthropogenic** | |  |  |  |  |  |
|  | Colonisation pressure | total | log+1 |  | no. species introduced | GAVIA database |
|  | Time since introduction | earliest | log |  | years since first record | GAVIA database |
|  | Human population density | mean | log+1 | 1x1 km grid | persons per km2 | http://sedac.ciesin.columbia.edu/gpw |
|  | Human population density | median | log+1 | 1x1 km grid | persons per km2 | http://sedac.ciesin.columbia.edu/gpw |
|  | Human footprint | median |  | 1x1 km grid | Human Influence Index (HII) | http://dx.doi.org/10.7927/H4M61H5F |
|  | Distance to city | median | log | 30 arc seconds | minutes of travel time | http://bioval.jrc.ec.europa.eu/products/gam |
|  | Distance to historic port | median | square root |  | kilometres to port | <http://pendientedemigracion.ucm.es/info/cliwoc/cliwoc15.htm> |
| **Environmental** | |  |  |  |  |  |
|  | Native species richness | total | square root |  | no. native species | ADHoC database |
|  | Elevation | median | log | 30 arc seconds | metres above sea level | http://www.worldclim.org |
|  | Elevation | range | square root | 30 arc seconds | metres above sea level | http://www.worldclim.org |
|  | Temperature | median |  | 30 arc seconds | degrees centigrade | http://www.worldclim.org |
|  | Temperature | minimum |  | 30 arc seconds | degrees centigrade | http://www.worldclim.org |
|  | Temperature | maximum |  | 30 arc seconds | degrees centigrade | http://www.worldclim.org |
|  | Temperature | range | square root | 30 arc seconds | degrees centigrade | http://www.worldclim.org |
|  | Precipitation | median | square root | 30 arc seconds | millimetres | http://www.worldclim.org |
|  | Habitat complexity | 8 grid cells |  | 300 m | number of landcover types | http://www.esa-landcover-cci.org/?q=node/158 |
|  | Habitat complexity | 24 grid cells |  | 300 m | number of landcover types | http://www.esa-landcover-cci.org/?q=node/158 |
