# Supplementary material for: The Global Distribution and Drivers of Alien Bird Species Richness
Source: PLoS Biol. 2017 Jan 12;15(1):e2000942. doi: 10.1371/journal.pbio.2000942 (PMC5230740; doi:10.1371/journal.pbio.2000942)
Supplement: S3 Table — The number of species introduced from a family in the first quartile (historical) and fourth quartile (modern), the total number of species in the family (Total) [46], and the probability (Psim calculated using simulations; see Methods) of observing as many or more introductions (or fewer introductions for those in the shaded areas) from that family given the number of species in the family and the proportion of the world’s bird species that have been introduced. The families shown are those with probabilities that are significantly lower than expected (α = 0.05), once a sequential Bonferroni correction for multiple statistical tests [72] has been applied. (DOCX) [file pbio.2000942.s008.docx]

|  | No. spp. Introduced | |  | *P* sim | |
| --- | --- | --- | --- | --- | --- |
| Family | Historical | Modern | Total | Historical | Modern |
| Anatidae | 21 | 44 | 173 | <0.0001 | <0.0001 |
| Estrildidae | 15 | 22 | 138 | <0.0001 | <0.0001 |
| Phasianidae | 40 | 17 | 183 | <0.0001 | <0.0001 |
| Columbidae | 19 | 13 | 320 | <0.0001 |  |
| Fringillidae | 18 | 10 | 179 | <0.0001 |  |
| Odontophoridae | 6 | 3 | 31 | <0.0001 |  |
| Anseranatidae | 0 | 1 | 1 |  | <0.0001 |
| Ciconiidae | 0 | 5 | 19 |  | <0.0001 |
| Phoenicopteridae | 1 | 4 | 6 |  | <0.0001 |
| Ploceidae | 6 | 12 | 106 |  | <0.0001 |
| Psittacidae | 16 | 76 | 374 |  | <0.0001 |
| Sturnidae | 9 | 20 | 115 |  | <0.0001 |
| Gruidae | 0 | 4 | 15 |  | 0.0002 |
| Tyrannidae | 0 | 0 | 416 | 0.0002 | <0.0001 |
| Trochilidae | 0 | 0 | 338 | 0.0002 | 0.0002 |
| Sylviidae | 2 | 0 | 299 |  | 0.0002 |
