# Supplementary material for: The Global Distribution and Drivers of Alien Bird Species Richness
Source: PLoS Biol. 2017 Jan 12;15(1):e2000942. doi: 10.1371/journal.pbio.2000942 (PMC5230740; doi:10.1371/journal.pbio.2000942)
Supplement: S4 Table — Prop. historic = the proportion of species introduced in the first quartile of bird introductions (historical era) from each biogeographic region; Prop. modern = the proportion of species introduced in in the fourth quartile of bird introductions (modern era) from each biogeographic region. The numbers (and proportions) of species sourced from each biogeographic realm during the two time periods differed significantly (chi2 = 22.88, p <0.001). (DOCX) [file pbio.2000942.s009.docx]

| Realm | Historical | Modern | Prop. historic | Prop. modern |
| --- | --- | --- | --- | --- |
| Afrotropical | 34 | 59 | 0.15 | 0.20 |
| Australasian | 31 | 40 | 0.14 | 0.13 |
| Indo-Malay | 36 | 65 | 0.16 | 0.22 |
| Nearctic | 23 | 18 | 0.10 | 0.06 |
| Neotropical | 24 | 57 | 0.11 | 0.19 |
| Oceanic | 4 | 3 | 0.02 | 0.01 |
| Palearctic | 73 | 56 | 0.32 | 0.19 |
