# Supplementary material for: The Global Distribution and Drivers of Alien Bird Species Richness
Source: PLoS Biol. 2017 Jan 12;15(1):e2000942. doi: 10.1371/journal.pbio.2000942 (PMC5230740; doi:10.1371/journal.pbio.2000942)
Supplement: S5 Table — S.E. = standard error for the variable. ∑CPO = the sum of the probabilities of each data point given the model. For comparison, fitting an intercept only model gives wAIC = –11,610.6 and CPO = 4,457.5. (DOCX) [file pbio.2000942.s010.docx]

|  | **Estimate ± S.E.** | |  | ***∑CPO*** |
| --- | --- | --- | --- | --- |
| **Predictor** | **Linear** | **Quadratic** | **AIC** |  |
| Colonization pressure | 0.053932 ± 0.00119 | -0.000341 ± 0.000013 | -13198 | 5370.7 |
| Native richness | 0.002629 ± 0.00014 | -0.000002 ± 1.71E-7 | -12111 | 4670.8 |
| Time since introduction | 0.0037270 ± 7.16E-8 | -0.000001 ± 0.000208 | -11964 | 4575.9 |
| Distance to historic port | -0.000449 ± 0.00008 |  | -11649 | 4469.6 |
| Precipitation | 0.000149 ± 0.00003 | -2.8E-8 ± 5.01E-9 | -11647 | 4466.8 |
| Elevation (range) | 0.000455 ± 0.0003 | -0.000003 ± 9.79E-7 | -11608 | 4454.1 |
| Temperature (median) | 0.000081 ± 0.00001 | -1.1E-8 ± 2.12E-9 | -11606 | 4481.7 |
| Habitat complexity | 0.006394 ± 0.00661 |  | -11595 | 4454.6 |
| Human footprint index | 0.003595 ± 0.00095 |  | -11465 | 4461.4 |
