# Supplementary material for: The Global Distribution and Drivers of Alien Bird Species Richness
Source: PLoS Biol. 2017 Jan 12;15(1):e2000942. doi: 10.1371/journal.pbio.2000942 (PMC5230740; doi:10.1371/journal.pbio.2000942)
Supplement: S6 Table — The shaded column indicates the selected predictors and values for the minimum adequate model using all of the data. Goodness-of-fit was calculated with Pearson’s correlation coefficients between the response variables and the fitted values of the models (i.e. pseudo R2). Average RMSE of holdout models = 0.735 (i.e. overall predictive accuracy = e0.725 = 2.08 species per grid cell). MAM = minimum adequate model. RMSE = root mean squared error. AIC = Akaike’s Information Criterion. (DOCX) [file pbio.2000942.s011.docx]

| **Predictors** | **MAM of full data** | **Hold-out Afrotropical** | **Hold-out Australasian** | **Hold-out Indo-Malay** | **Hold-out Nearctic** | **Hold-out Neotropical** | **Hold-out Palearctic** |
| --- | --- | --- | --- | --- | --- | --- | --- |
| Colonisation pressure | 1206.8 | 26.9 | 96.9 |  | 132.3 | 89.5 |  |
| Colonisation pressure ² | 381 | 58.2 | 87.7 |  | 9.4 | 8.5 |  |
| Time since introduction | 153.4 |  |  | 92 |  |  | 62.4 |
| Time since introduction² | 43.9 |  |  | 5.4 |  |  | 92.8 |
| Native species richness | 320.2 | 145.2 | 57.7 | 321.5 | 5.5 | 146.7 | 73.8 |
| Native species richness² | 123 | 6.3 | 38.7 |  | 12.8 | 4.1 | 72.4 |
| Distance to historic port | 4.9 |  |  | 9.2 | 8.8 |  | 9.3 |
| Distance to historic port^2^ |  |  |  | 26.1 | 16.2 |  | 15.8 |
| Precipitation |  | 6.3 |  | 8.8 |  |  | 4.6 |
| Precipitation ² |  | 5.6 | 6 | 31 |  |  | 4.1 |
| Elevational range |  | 4.5 |  |  |  | 5.1 |  |
| Temperature median |  |  |  |  |  |  |  |
| Temperature median ² |  |  |  |  |  |  |  |
| Habitat complexity |  |  |  |  | 6.7 |  |  |
| **RMSE** | 0.066 | 0.574 | 1.024 | 0.836 | 0.69 | 0.537 | 0.746 |
| **Goodness-of-fit** | 0.986 | 0.981 | 0.981 | 0.991 | 0.984 | 0.979 | 0.986 |
