# Supplementary material for: The Global Distribution and Drivers of Alien Bird Species Richness
Source: PLoS Biol. 2017 Jan 12;15(1):e2000942. doi: 10.1371/journal.pbio.2000942 (PMC5230740; doi:10.1371/journal.pbio.2000942)
Supplement: S7 Table — Parameter estimates are given fitting a Gaussian random field to the data to approximate the patterns of spatial autocorrelation in a Bayesian additive regression model inferred using INLA. wAIC = –12,449, conditional predictive ordinate (CPO) measure of fit = 4,797.2. For comparison, fitting an intercept only model gives wAIC = –11,610.6 and CPO = 4,457.5. S.E. = standard error. n = 10,258 grid cells. (DOCX) [file pbio.2000942.s012.docx]

| **Parameter** | | **Estimate** | **S.E.** | **ΔwAIC** | |
| --- | --- | --- | --- | --- | --- |
| **Intercept** | |  |  |  |  |
|  | | –1.12 | 0.16 | 0 |  |
| **Anthropogenic** | |  |  |  |  |
|  | Time since introduction | 0.0036 | 0.0003 | 282.7 |  |
|  | (Time since introduction)^2^ | –1.3E–6 | 7E–8 | 32.1 |  |
|  | Distance to historic port | –1.9E–7 | 7E–8 | 61.9 |  |
| **Environmental** | |  |  |  |  |
|  | Native Species Richness | 0.0025 | 0.00013 | 381.2 |  |
|  | (Native Species Richness)² | –2.0E–6 | 1.7E–7 | 119.7 |  |
|  | Precipitation | 8E-5 | 2.5E–5 | 6.1 |  |
|  | (Precipitation)^2^ | –2E-8 | 0 | 14.4 |  |
