# Supplementary material for: The Global Distribution and Drivers of Alien Bird Species Richness
Source: PLoS Biol. 2017 Jan 12;15(1):e2000942. doi: 10.1371/journal.pbio.2000942 (PMC5230740; doi:10.1371/journal.pbio.2000942)
Supplement: S8 Table — r is above the diagonal and P is below it. (DOCX) [file pbio.2000942.s013.docx]

|  | native richness | colonisation  pressure | time since  introduction | elevation (median) | elevation (range) | temperature °C  (median) | temperature °C  (max.) | temperature °C  (min.) | temperature °C  (range) | population  (mean) | population  (median) | distance to city | human footprint | habitat complexity  (8 cells) | habitat complexity  (24 cells) | distance to  historic port | precipitation  (median) |
| --- | --- | --- | --- | --- | --- | --- | --- | --- | --- | --- | --- | --- | --- | --- | --- | --- | --- |
| native richness |  | -0.21 | -0.19 | 0.09 | 0.08 | 0.45 | 0.34 | 0.44 | -0.41 | 0.16 | 0.15 | -0.11 | 0.09 | 0.11 | 0.12 | -0.08 | 0.51 |
| colonisation pressure | 0.000 |  | 0.29 | -0.13 | 0.09 | 0.13 | 0.00 | 0.18 | -0.24 | 0.30 | 0.26 | -0.25 | 0.32 | -0.05 | -0.05 | -0.26 | 0.12 |
| time since introduction | 0.000 | 0.000 |  | -0.12 | -0.08 | 0.09 | 0.00 | 0.14 | -0.14 | 0.10 | 0.04 | -0.32 | 0.20 | -0.02 | 0.00 | -0.23 | 0.06 |
| elevation (median) | 0.000 | 0.000 | 0.000 |  | 0.63 | -0.24 | -0.20 | -0.26 | 0.26 | -0.18 | -0.19 | 0.13 | -0.23 | 0.14 | 0.15 | 0.20 | -0.28 |
| elevation (range) | 0.000 | 0.000 | 0.000 | 0.000 |  | -0.10 | -0.25 | -0.05 | -0.05 | 0.16 | 0.15 | 0.00 | 0.04 | 0.11 | 0.13 | -0.19 | -0.01 |
| temperature °C (median) | 0.000 | 0.000 | 0.000 | 0.000 | 0.000 |  | 0.84 | 0.97 | -0.83 | 0.40 | 0.40 | -0.37 | 0.35 | -0.08 | -0.09 | -0.34 | 0.51 |
| temperature °C (max.) | 0.000 | 0.791 | 0.739 | 0.000 | 0.000 | 0.000 |  | 0.71 | -0.41 | 0.27 | 0.27 | -0.37 | 0.24 | -0.07 | -0.09 | -0.08 | 0.23 |
| temperature °C (min.) | 0.000 | 0.000 | 0.000 | 0.000 | 0.000 | 0.000 | 0.000 |  | -0.93 | 0.41 | 0.41 | -0.35 | 0.37 | -0.08 | -0.09 | -0.42 | 0.57 |
| temperature °C (range) | 0.000 | 0.000 | 0.000 | 0.000 | 0.000 | 0.000 | 0.000 | 0.000 |  | -0.38 | -0.39 | 0.22 | -0.34 | 0.09 | 0.10 | 0.50 | -0.66 |
| population (mean) | 0.000 | 0.000 | 0.000 | 0.000 | 0.000 | 0.000 | 0.000 | 0.000 | 0.000 |  | 0.96 | -0.74 | 0.88 | 0.08 | 0.11 | -0.33 | 0.35 |
| population (median) | 0.000 | 0.000 | 0.000 | 0.000 | 0.000 | 0.000 | 0.000 | 0.000 | 0.000 | 0.000 |  | -0.67 | 0.86 | 0.08 | 0.11 | -0.32 | 0.36 |
| distance to city | 0.000 | 0.000 | 0.000 | 0.000 | 0.845 | 0.000 | 0.000 | 0.000 | 0.000 | 0.000 | 0.000 |  | -0.80 | -0.08 | -0.12 | 0.26 | -0.12 |
| human footprint | 0.000 | 0.000 | 0.000 | 0.000 | 0.000 | 0.000 | 0.000 | 0.000 | 0.000 | 0.000 | 0.000 | 0.000 |  | 0.06 | 0.09 | -0.32 | 0.30 |
| habitat complexity (8 cells) | 0.000 | 0.000 | 0.046 | 0.000 | 0.000 | 0.000 | 0.000 | 0.000 | 0.000 | 0.000 | 0.000 | 0.000 | 0.000 |  | 0.81 | -0.01 | 0.02 |
| habitat complexity (24 cells) | 0.000 | 0.000 | 0.678 | 0.000 | 0.000 | 0.000 | 0.000 | 0.000 | 0.000 | 0.000 | 0.000 | 0.000 | 0.000 | 0.000 |  | -0.02 | 0.04 |
| distance to historic port | 0.000 | 0.000 | 0.000 | 0.000 | 0.000 | 0.000 | 0.000 | 0.000 | 0.000 | 0.000 | 0.000 | 0.000 | 0.000 | 0.530 | 0.052 |  | -0.37 |
| precipitation (median) | 0.000 | 0.000 | 0.000 | 0.000 | 0.379 | 0.000 | 0.000 | 0.000 | 0.000 | 0.000 | 0.000 | 0.000 | 0.000 | 0.017 | 0.000 | 0.000 |  |
